# Impact of developmental state, p53 status, and interferon signaling on glioblastoma cell response to radiation and temozolomide treatment

**DOI:** 10.1371/journal.pone.0315171

**Published:** 2025-02-07

**Authors:** Artem Berezovsky, Oluwademilade Nuga, Indrani Datta, Kimberly Bergman, Thais Sabedot, Katherine Gurdziel, Susan Irtenkauf, Laura Hasselbach, Yuling Meng, Claudius Mueller, Emanuel F. . Petricoin, Stephen Brown, Neeraja Purandare, Sidhesh Aras, Tom Mikkelsen, Laila Poisson, Houtan Noushmehr, Douglas Ruden, Ana C. deCarvalho

**Affiliations:** 1 Department of Neurosurgery, Henry Ford Health, Detroit, Michigan, United States of America; 2 Department of Oncology, Wayne State University, Detroit, Michigan, United States of America; 3 Department of Pharmacology, Wayne State University, Detroit, Michigan, United States of America; 4 Institute of Environmental Health Sciences, Wayne State University, Detroit, Michigan, United States of America; 5 Center for Applied Proteomics and Molecular Medicine, George Mason University, Manassas, Virginia, United States of America; 6 Department of Radiation Oncology, Henry Ford Health, Detroit, Michigan, United States of America; 7 Precision Medicine Program, Henry Ford Health, Detroit, Michigan, United States of America; 8 Department of Public Health, Henry Ford Health, Detroit, Michigan, United States of America; 9 Department of Obstetrics and Gynecology, School of Medicine, Wayne State University, Detroit, Michigan, United States of America; 10 Department of Physiology, College of Human Medicine, Michigan State University, East Lansing, Michigan, United States of America; Bauer Research Foundation, UNITED STATES OF AMERICA

## Abstract

Glioblastoma (GBM) tumors exhibit extensive genomic, epigenomic, and transcriptional diversity, with significant intratumoral heterogeneity, complicating standard treatment approaches involving radiation (RT) and the DNA-alkylating agent temozolomide (TMZ). In this study, we employed an integrative multi-omics approach, including targeted proteomics, transcriptomics, genomics, and DNA methylation profiling, to investigate the response of a representative panel of GBM patient-derived cancer stem cells (CSCs) to astrocytic differentiation and RT and TMZ treatments. Differentiated CSC progenies retained the expression of key stemness genes and survival pathways, while activating the BMP-Smad signaling pathway and upregulating extracellular matrix components. This was associated with increased resistance to TMZ, though not to RT, across all models. We identified TP53 status as a critical determinant of transcriptional response to both RT and TMZ, which was also modulated by the differentiation state and treatment modality in wildtype (wt) p53 GBM cells. Both mutant and wt p53 models exhibited significant activation of the DNA-damage associated interferon (IFN) response in CSCs and differentiated cells, implicating this pathway in the GBM response to therapy. We observed that activation of NF-κB was positively correlated with the levels of O-6-methylguanine-DNA methyltransferase (MGMT) protein, a direct DNA repair enzyme leading to TMZ resistance, regardless of MGMT promoter methylation status, further supporting the clinical potential for inhibition of NF-kB signaling in GBM treatment. Our integrative analysis of the impact of GBM cell developmental states, in the context of genomic and molecular diversity of patient-derived models, provides valuable insights for pre-clinical studies aimed at optimizing treatment strategies.

## Introduction

Glioblastoma (GBM), classified as World Health Organization (WHO) Grade 4 isocitrate dehydrogenase (IDH)-wildtype astrocytoma, remains the most aggressive and prevalent primary brain tumor in adults [1]. Since 2005, standard of care treatment of GBM includes maximal safe surgical resection [2], followed by ionizing radiation therapy (RT) and the brain-penetrant DNA-alkylating agent temozolomide (TMZ) [3]. Surgery alone is not curative [2] and TMZ provides a modest, significant increase in median overall survival in patients with newly diagnosed GBMs when combined with RT compared to RT alone [4]. TMZ sensitivity in newly diagnosed GBMs is modulated by the expression of DNA repair protein, O-6-methylguanine-DNA methyltransferase (MGMT). The silencing of MGMT through promoter hypermethylation is a predictive marker of TMZ sensitivity for newly diagnosed GBMs [5], while MGMT expression is also regulated by DNA methylation independent mechanisms [6,7]. Most GBM patients have known actionable therapeutical targets [8], clinical trials for targeted therapies have not demonstrated sufficient efficacy in improving overall and progression-free survival [9].

Intra-tumoral heterogeneity (ITH) is a key factor contributing to GBM resistance to treatment and high recurrence rate. Genomically-defined subclonal populations presenting differential sensitivity to pharmacological agents can arise in post-treatment recurrent GBMs [10]. Another contributor to ITH is oncogene amplification in extrachromosomal DNA (ecDNA), as we previously reported cases of low frequency ecDNA in newly diagnosed GBM exhibiting a notable increase in prevalence at recurrence following treatment [11]. Non-genetic factors also contribute to ITH, such as the high degree of plasticity of GBM cells, reflected in the ability to transition into a continuum of cell states analogous to neural development, from neural stem/progenitor-like to differentiated astrocyte-like cells, as shown by bulk tumor RNA sequencing (seq) deconvolution and single cell RNAseq analyses [12]. ITH involves dynamic shifts in subclonal composition in response to treatment and to changes in the microenvironment in GBM [13,14]. Thus, it is important to consider ITH when designing preclinical studies to test optimization of treatment efficacy for GBMs.

Tumor cell subpopulations exhibiting cancer stem cell-like (CSC) properties contribute to another layer of ITH through developmental cell state plasticity [15]. Long-term self-renewal in culture and the ability to differentiate and recapitulate the original tumor upon orthotopic implantation in immunocompromised rodents are defining attributes of cancer stem cells (CSCs) [16,17]. These properties make CSCs invaluable for developing GBM patient-derived models for pre-clinical studies. An established strategy to select for GBM CSC from surgical samples involves culturing dissociated tumor cells in selective serum-free media, originally formulated for the isolation of mouse neural progenitor cells, resulting in “neurosphere” cultures [18,19]. In recent years, GBM neurosphere cultures have been extensively validated as a renewable source of patient-derived neoplastic cells retaining remarkable genomic fidelity to the original tumor [11,20,21]. These neurosphere/CSC cultures are amenable to in vitro biological and experimental therapeutics studies [22] and are suitable for validation in xenografts. To enhance the translational applicability of in vitro investigations aimed at optimizing treatment efficacy in GBMs, it is imperative to consider the influence of patient genomic diversity and intra-tumoral phenotypic variability. Ultimately, the diversity in genomic and epigenomic drivers among GBM patients, along with the additional variability associated with dynamic ITH, converge on the deregulation of key oncogenic signaling involving receptor tyrosine kinases (RTK), phosphoinositide 3-kinases (PI3K)- protein kinase B (AKT)- mammalian target of rapamycin (mTOR) and mitogen-activated protein kinase (MAPK) pathways. These pathways are deregulated in 90% of GBM patients [23], and cross activation of PI3K and MAPK contribute to several aspects of GBM malignancy [24].

The objective of this study is to investigate the contributions of somatic genomic alterations in conjunction with GBM cellular developmental states to the modulation of key signaling pathways and response to TMZ and RT. Employing targeted proteomics, we assessed changes in the steady-state levels of key oncogenic signaling components, following a 2-week exposure of CSCs to astrocytic differentiation (SDCs), growth factor withdrawal, or traditional culture media supplemented with 10% FBS. in a genomically-diverse panel of CSCs derived from newly diagnosed GBM patients [11,25,26]. The representation of various genomic backgrounds and external stimuli in the targeted proteomics dataset provided insights into the complex signaling networks co-activated in GBMs, while uncovering a strong positive correlation between nuclear factor-kappa B (NF-κB) activation and MGMT protein expression, regardless of MGMT promoter methylation status. Some of the signaling and transcriptional program adaptations in response to astrocytic differentiation of CSCs were shared among the genomically distinct models, including BMP-Smad pathway activation, decrease in cholesterol biosynthesis, increase in extracellular matrix (ECM) production, and retention of the expression of a subset of stemness genes. Other CSC differentiation mediated alterations were model specific, such as activation of the interferon (IFN) response. Here, we show that differentiation of CSCs increases resistance to TMZ but not to RT, and that transcriptional responses to these treatments are largely regulated by p53 in wildtype (wt) p53 GBM cells and modulated by cell differentiation state. In both mutant and wt p53 models, we observed prominent activation of DNA-damage associated IFN response. We show that modeling the differentiation of GBM CSCs in vitro provides a powerful platform to uncover the interaction of genomic landscape and cell state on signaling pathways and response to treatment.

## Results

### Alterations in key cell signaling pathways in glioblastoma cancer stem cells in response to astrocytic differentiation, growth factor withdrawal and traditional 10% FBS media

**Fig 1:**
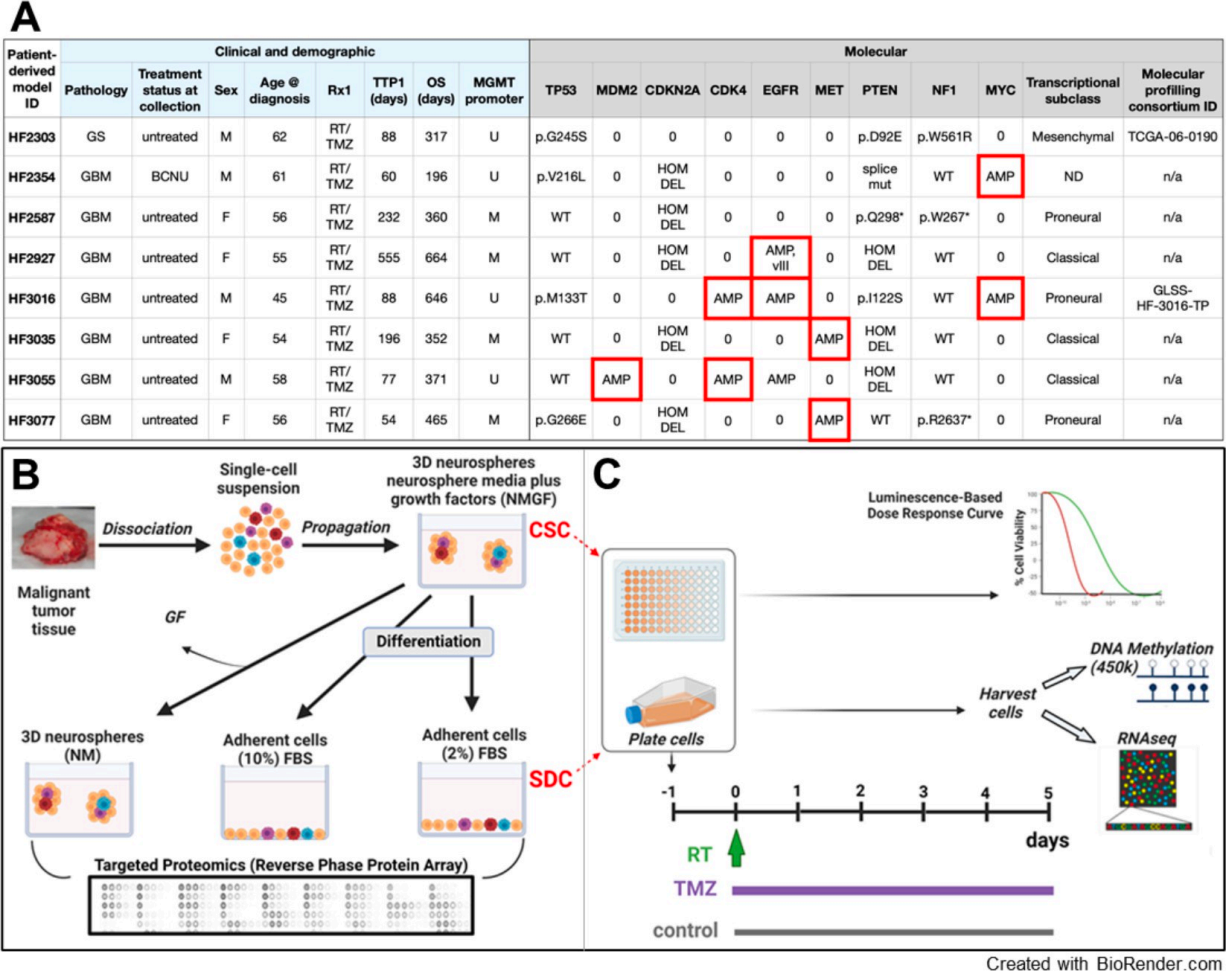
Glioblastoma patient-derived models and experimental design. **A)** Clinical and molecular data associated with the GBM patient-derived models. GS, Gliosarcoma; M/F, male/female; RT, radiation therapy; TMZ, temozolomide; Rx1, first-line therapy; TTP, time to progression; OS, overall survival; U/M, unmethylated/methylated MGMT promoter. Red outline denotes extrachromosomal (ecDNA) amplification. MDM2, Mouse double minute 2 homolog; CDKN2A, cyclin-dependent kinase inhibitor 2A; NF1, Neurofibromin 1; MYC family, MYC proto-oncogene, bHLH transcription factor; ND, not determined; AMP, amplification; mut, mutation; hom del, homozygous deletion; WT, wild type; 0, diploid. **B)** Schematic depicting neurosphere culture in NMGF media for selection and amplification of cancer stem cells (CSCs) from surgical specimens, followed by 2 weeks incubation in altered conditions: Withdrawal of growth factors (NM), and addition of 2% or 10% FBS. For the purposes of this study, serum-differentiated cells (SDC) are CSCs progeny cultured in NMGF supplemented with 2% FBS. The effect of the different culture conditions on cell signaling were compared by RPPA. **C)** The sensitivity of CSC and SDC to single dose radiation (RT) or 5-day TMZ was measured in three select models. Transcriptional and epigenomic reprograming in SDC vs CSC, and in response to treatment were evaluated by bulk RNAseq and 450k DNA methylation array. Panels B and C were generated in BioRender (Created in BioRender. Berezovsky, A. (2024) https://BioRender.com/n49q520).

**Fig 2:**
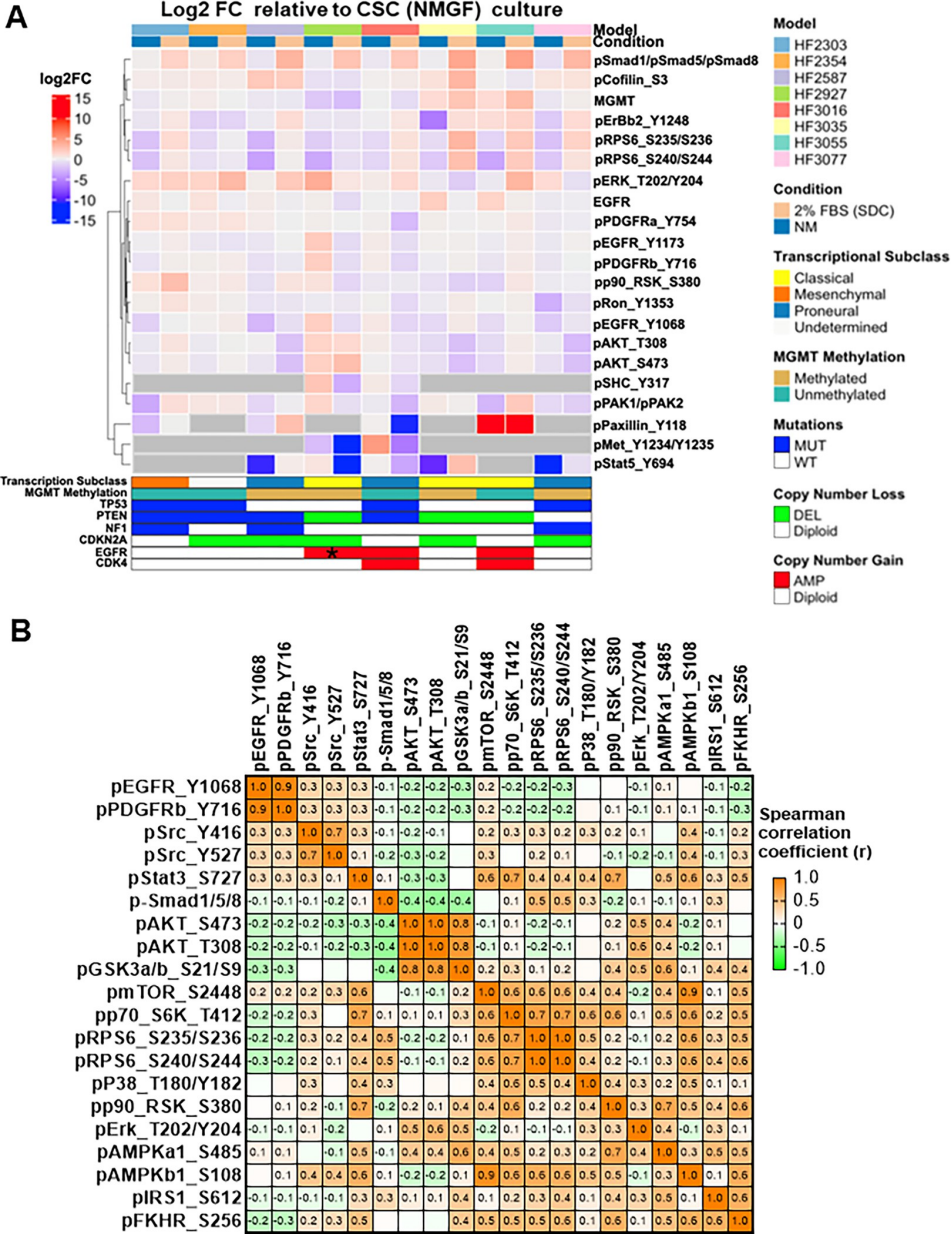
Targeted proteomics reveals common and cell specific alterations in key signaling CSCs in response to differentiation and identifies interdependencies in activation of key signaling mediators. **A)** Heatmap depicting log2 fold change (FC) relative to NMGF (CSC) for the top 5% most variable proteins/PTM. Each value represents mean log2FC for n = 3 RPPA measurements/group. Proteins/PTMs which were not detected under all three culture conditions were filtered out for each model (grey cells). MUT, mutation; WT, wild type; AMP, amplification; DEL, deletion **B)** Heatmap of Spearman correlation coefficient values comparing phosphorylation levels in key cell signaling mediators in 8 patient derived GBM models grown in 4 media conditions in triplicates.

CSCs derived from 8 newly diagnosed GBM patients, representing the main genomic drivers were selected for this study (Fig 1A). These CSC lines meet the requirement of the two defining stemness criteria: long term self-renewal and orthotopic tumor formation in immunocompromised mice [11,25,26]. FBS, a supplement supporting the culture of a variety of mammalian cells, contains hormones, growth factors, cell attachment factors and other nutrients [27]. Media supplemented with 10% FBS had been widely employed to propagate glioma cells in culture, prior to seminal data demonstrating genetic drift occurring from serial passaging under these conditions [28]. In contrast, 1–2% FBS added to defined media has been successfully used to differentiate human-induced pluripotent stem cells (hiPSC)-derived neural stem cells into astrocytic lineage [29,30]. Using this paradigm to model a differentiated phenotype in culture, CSCs growing in 3D cultures in defined neurosphere media, supplemented with, 20 ng/ml each epidermal (EGF) and basic fibroblast (bFGF) growth factors (NMGF), were differentiated into an astrocytic phenotype in NMGF supplemented with 2% FBS for 14 days. This differentiated CSC progeny is referred to here as “serum differentiated cells” (SDC). For comparison, we tested two additional growth conditions: growth factor-depleted neurosphere media (NM), or media supplemented with 10% FBS (10% FBS) (Fig 1B). After 2 weeks in culture, cell lysates were obtained and analyzed by Reverse Phase Protein Arrays (RPPA) in triplicates, as described [31,32]. The levels of 66 proteins or post-translational modifications (PTM) in the cell lysates were quantified (S1 Table). Pairwise comparisons between NMGF (CSC) culture and either NM or 2% FBS (SDC) for the 8 cell lines did not show a global shift in the levels of protein/PTMs levels (S1 Fig), but important patterns of alteration in specific signaling were observed. Phosphorylation (phospho) levels of serine (Ser)-residues in receptor-dependent r-Smad1/5/8, which are primarily activated by BMP ligand signaling, were notably upregulated in SDCs in relation to CSCs in all 8 GBM models (Fig 2A). Consistent with bone morphogenetic protein (BMP) ligands being present in FBS [33], increased levels of p-Smad1/5/8 were also observed in 10% FBS cultures for all models (S1 Table). The tumor suppressor phosphatase and tensin homolog (PTEN) function is frequently lost in GBMs through gene deletion and mutations, as seen in 7/8 GBM tumors represented in this study (Fig 1A), leading to activation of AKT [34]. AKT phosphorylation at both Y308 (by pyruvate dehydrogenase kinase 1 (PDK1)) and S473 (by mammalian target of rapamycin complex 2 (mTORC2)) was suppressed in the absence of growth factors (NM) for all lines, except for HF2927, which carries ligand independent epidermal growth factor receptor (EGFR) variant III (viii) amplification. In 2% FBS (SDCs), AKT phosphorylation was suppressed for HF2587, HF3035 and HF3077, and upregulated or unchanged in the remaining models (Fig 2A). In response to growth factor withdrawal, phosphorylation levels of S6 ribosomal proteins (RBS6), downstream of mTORC1, were decreased while phospho- extracellular signal-regulated kinases **(**ERK) increased for most CSC lines (Fig 2A). Phospho-EGFR (Y1173 and Y1068) levels were increased in response to growth factor withdrawal exclusively in the EGFRvIII line HF2927 (Fig 2A). Expression of MET proto-oncogene, receptor tyrosine kinase (cMET) was observed for all CSCs (S1 Table), but in the absence of MET ligand (hepatocyte growth factor) in the media, only HF2927 and HF3016 presented modest levels of MET activation, which was suppressed in 2% FBS (Fig 2A).

We used this unique targeted proteomics dataset, integrating various genomic backgrounds and culture conditions, to further investigate the signaling networks in glioblastoma. The Spearman correlation of key proteins and post-translational modifications levels in all 96 samples (8 GBM lines in 4 media conditions in triplicate) was calculated and displayed in the heatmap (Fig 2B). Glycogen synthase kinase-3 (GSK3)α/β is a multifunctional kinase frequently inhibited by phosphorylation downstream of PI3K/AKT, mTORC1 or MAPK signaling. Here, we observed that phosphorylation of GSK3α/β at S21/S9 was strongly correlated with AKT activation (Fig 2B). While mTORC1 is activated by AKT [35], in this dataset AKT activation did not correlate with markers of mTORC1 signaling, such as mTOR phosphorylation at the C-terminus S2448 residue [36], p70 ribosomal protein S6 kinase 1 (S6K) phosphorylation at T412, or phosphorylation of RBS6 [37] (Fig 2B). On the other hand, phosphorylation of AMP-activated protein kinase (AMPK) subunit β1 at S108 was highly correlated with mTORC1 activation (Fig 2B). AMPK αβɣ heterotrimeric complex is activated in response to low oxygen, low nutrients, and DNA damage, playing important roles in cancer [37]. AMPK is inactivated by AKT phosphorylation of AMPKα1 at S485 residue, while phosphorylation at AMPKβ1 S108 sensitizes AMPK to agonists [38], having a function in pro-survival pathways [39]. SRC proto-oncogene, non-receptor tyrosine kinase (Src) is a node of convergence for receptor mediated signaling pathways, activating substrates in the MAPK, JAK/Stat3, and PI3K/AKT pathways to promote tumor cell survival, proliferation and invasion in various cancers, including GBMs [40]. Src activity is inhibited by phosphorylation of Y527 by C-terminal Src kinase (CSK) and regulated by dephosphorylation by protein tyrosine phosphatases (PTPs), leading to conformational change and autophosphorylation of Y416 [41]. Levels of inhibitory Src phospho-Y527 were elevated in HF2354, HF2587, HF3016 and HF3077 relative to other CSC lines while the levels of Src phospho-Y416 were less variable among CSC lines and media conditions (S1 Table) and positively correlated with Src phospho-Y527 (r = 0.705, CI: 0.584 to 0.796), but not with phospho- signal transducer and activator of transcription 3 (Stat3 S727), a canonical downstream signal transducer (Spearman correlation, r = 0.302, CI: 0.1026 to 0.4788) (Fig 2B). This bi-phosphorylation of Src has been previously reported to reflect an intermediate state between open and closed kinase conformations with blockage of the SH3 domain leading to limited Stat3 phosphorylation activity, suggesting that additional stimulatory factors are required for downstream signaling [42]. Phospho-Stat3 was positively correlated with mTORK1 (phospho-mTOR, phospho-SK6) and MAPK (phospho-RSK) signaling (Fig 2B). Signaling through insulin like growth factor 1 receptor (IGF1R) and insulin receptor (IR) promotes cancer cell proliferation, survival, and treatment resistance in diverse malignancies. Upon ligand binding, IGF1R homodimer or IGF1R/IR heterodimer receptors recruit insulin receptor substrate (IRS) adaptor protein to transduce downstream signaling through binding of src-homology-2 (SH2) proteins, such as p85 to activate PI3K and growth factor receptor bound protein 2 (GRB2) to activate ERK [43]. All four media conditions tested here contain insulin at a final concentration of 50 μg/ml (N2 supplement), which has been shown to activate IGF1R, although at a lower rate than the canonical ligand IGF2 [44], while the FBS supplemented media contains additional insulin and IGF1/2 ligands [27]. Consistently, activation of IGF1R (phospho-Y1135/Y1136) /IR (phospho-Y1150/Y1151) presented little variation among GBM lines and culture conditions (S1 Table). mTORC1 phosphorylates IRS1 at S616 residue leading to the adaptor turnover [45], but p-IRS1 did not correlate with mTORC1 activation. Instead, a strong correlation of p-IRS1 with inactivating phosphorylation of forkhead box protein O1 (FOXO1/FKHR) on S256, which is attributed to AKT [46], was observed (Fig 2B). This targeted proteomics analysis using genomically diverse patient samples in different culture conditions reveled a complex pattern in the integration of signaling cascade nodes, likely reflecting deregulation of multiple oncogenic pathways observed in GBMs.

**Fig 3:**
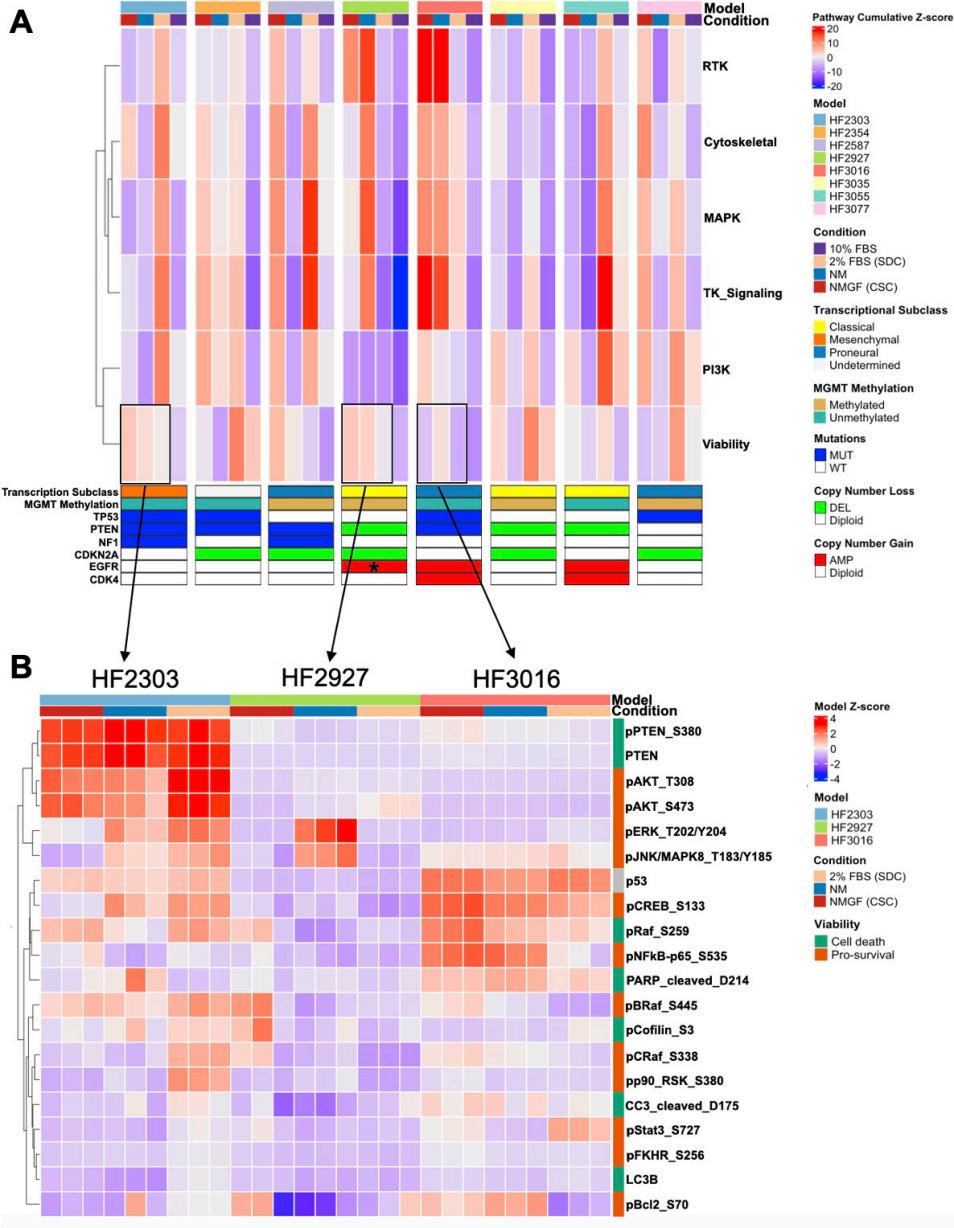
Comparative targeted proteomics of CSCs growing in 4 different conditions. **A)** Heatmap depicting mean signaling pathway activation for triplicate samples in 4 culture conditions, for CSCs derived from 8 patients with diverse genomic landscape, transcriptional subclass and MGMT promoter methylation. Hierarchical clustering of rows was based on Spearman correlation analysis. **B)** Heatmap for protein/PTM z-scores involved in cell death and survival signaling for 3 models grown in NMGF (CSC), NM and 2% FBS (SDC), in triplicate (boxes in (A)).

Z-scores were calculated for each protein/PTM based on its distribution across all 8 models and 4 media conditions, and cumulative z-scores were determined for key signaling pathways and cellular functions (S1 Table). Interestingly, we observed a remarkable pattern of inhibition for key signaling pathway and functions in 10% FBS, in contrast with 2% FBS (SDC) cultures (Fig 3A), further corroborating the inadequacy of culturing GBM cells in 10% FBS [28,47]. Receptor tyrosine kinase activation (“RTK”) index, based on the levels of Tyr phosphorylation corresponding to activation of 11 RTKs (S1 Table), decreased in 2% FBS (SDCs) relative to CSCs in HF2927 and HF3016, the only 2 models harboring EGFR amplification on extrachromosomal DNA (ecDNA) [11], and increased in the remaining 6 GBM models (Fig 3A). The levels of EGFR were 10-fold higher for HF2927 CSCs, which also carries the EGFRvIII variant (Fig 1A), relative to HF3016, while the levels of phospho-EGFR at carboxy-terminal Y1068 and Y1173 residues were comparable for these 2 CSC lines (Fig 3A). Interestingly, EGF withdrawal for 2 weeks (NM) led to a 4-fold increase in the levels of EGFR activation in HF2927 (S1 Table, Fig 2A), suggesting an inhibitory effect of long-term exposure to EGF ligand on EGFRvIII. RTK activation correlated with downstream RTK signaling pathway (“TK_Signaling”), which includes both MAPK and PI3K/Akt/mTOR (“PI3K”) pathways (Fig 3A). As part of RTK signaling convergence, SHC Adaptor Protein 1 (SHC1) binds to phosphorylated EGFR Y1173, which is further phosphorylated on Y239 and Y317 resulting in recruitment of Grb2 and downstream activation of MAPK and PI3K/AKT pathways [46]. Phospho-Shc1 (Y317) was only observed for HF2927 and HF3016 CSCs, and in both cases upregulated upon growth factor withdrawal (NM) and downregulated in the presence of FBS, following the pattern of EGFR activation (S1 Table, Fig 2A). MAPK signaling activation and cytoskeletal function, consisting of regulation of actin and microtube dynamics (S1 Table), were maintained or increased in SDCs for all models except HF2927 and HF3016 (Fig 3A), analogous to what we observed for the RTK index. The PI3K/AKT/mTOR pathway activation score was low for HF2927 CSCs, relative to the other CSC lines, and remained low in NM and 2% FBS, despite genomic loss of PTEN, EGFRvIII amplification, and high level of EGFR activation (Fig 3A). PI3K/AKT/mTOR activation index was higher for the other 7 CSCs, maintained in SDCs, and decreased in the absence of growth factors (Fig 3A).

Since several of these signaling pathways are involved in cell survival and proliferation, we established a cell “viability index” measuring the net result from the sum of the level of protein/PTM associated with cell survival and proliferation minus the level of markers associated with programed cell death (S1 Table). We observed that the viability index varied among CSC lines and was not impacted by culture conditions in the same pattern, decreasing in SDC in half of the models and increasing in the other half (Fig 3A). Three models, HF2303, HF2927 and HF3016, representing each of the 3 transcriptional subclasses, diverse genomic features (Fig 1A), and a range of viability index values, were selected for further characterization. Levels of individual protein/PTM associated with cell death and survival are shown (Fig 3B). High PTEN expression was observed in HF2303, harboring pathogenic p.D92E mutation in the WPD loop [48], corresponding to a high level of phosphorylation at S380 in the carboxy terminal tail, a crucial post-translational modification for blocking proteasomal degradation [49]. High levels of AKT activation observed for HF2303 CSCs were further increased in SDCs, indicating that the p.D92E mutation impairs PTEN ability to inhibit AKT. Conversely, PTEN levels were very low in HF3016, which carries a pathogenic mutation in the P-loop, p.I122S [48], comparable to PTEN-null HF2927 (Fig 3B). Increased levels of phospho-ERK and phospho- c-Jun N-terminal kinase (JNK) were observed for HF2303 and HF2927 in response to growth factor withdrawal (NM), and in HF2303, the increase was also observed in SDCs, relative to CSCs. The levels of p53 protein were consistent with the mutation status for these lines (Fig 3B). Thus, our comparative analysis confirms that at the individual protein/PTM levels, differences in pathways regulating cell survival and death in untreated GBM cells are largely determined by the genomic landscape, with cell state playing a lesser role.

### CSC presents increased sensitivity to temozolomide but not to radiation compared to their SDC progeny

**Fig 4:**
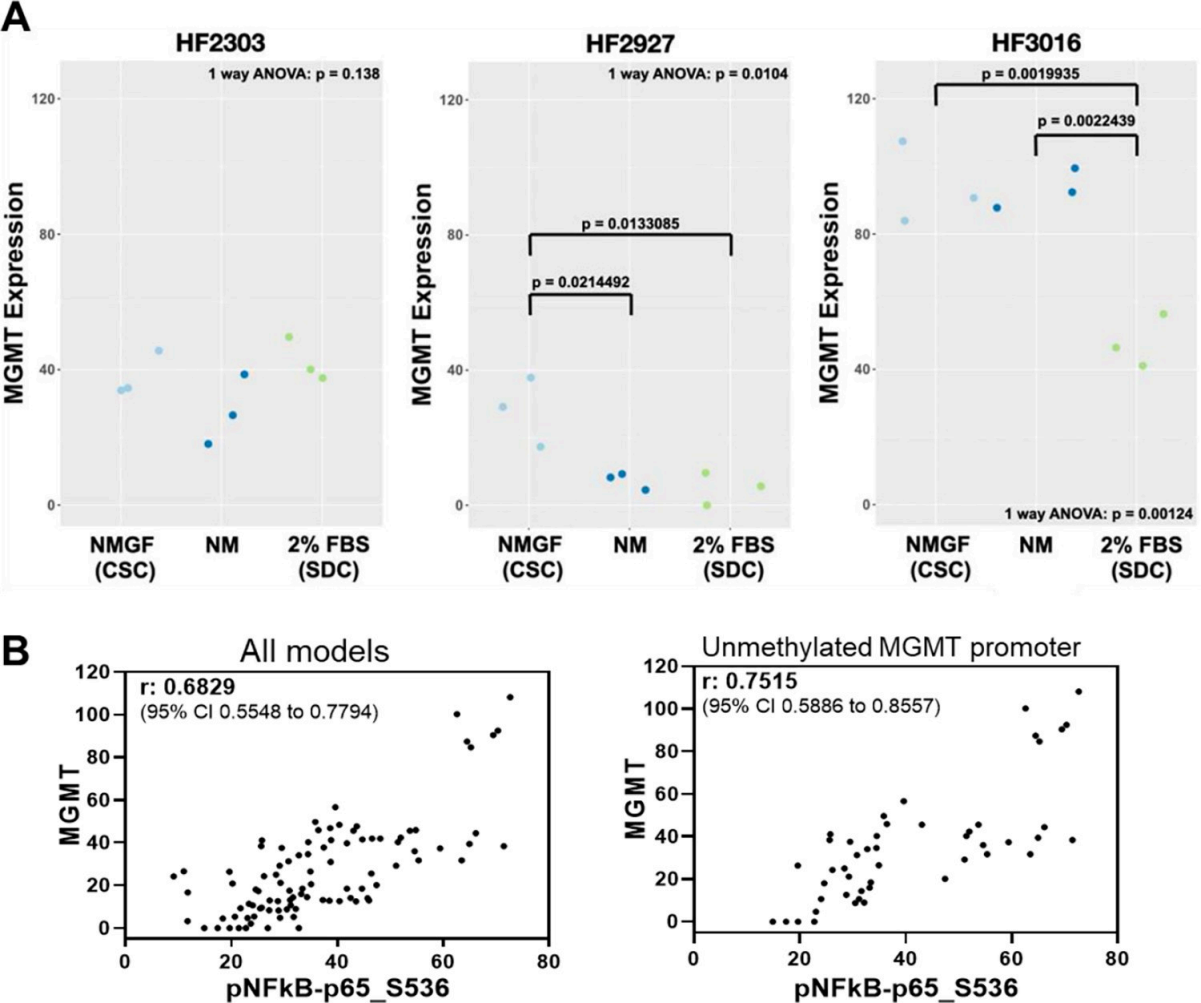
The impact of culture conditions on MGMT protein expression is cell line specific and correlates with NF-κB activation. **A)** MGMT protein levels measured by RPPA for HF2303, HF2927 and HF3016, in biological triplicates (S1 Table). P-values for 1-way ANOVA and post-hoc TukeyHSD test for comparison of the effect of culture conditions on MGMT protein expression are shown. Pairwise comparison between culture conditions was only significant for HF2927 and HF3016. **B)** Correlation of the levels of MGMT and phospho-NFκB (S536), for all 8 GBM models and culture conditions (left panel) and for the 4 models presenting unmethylated MGMT promoter (right panel). Spearman r and 95% CI are shown.

**Fig 5:**
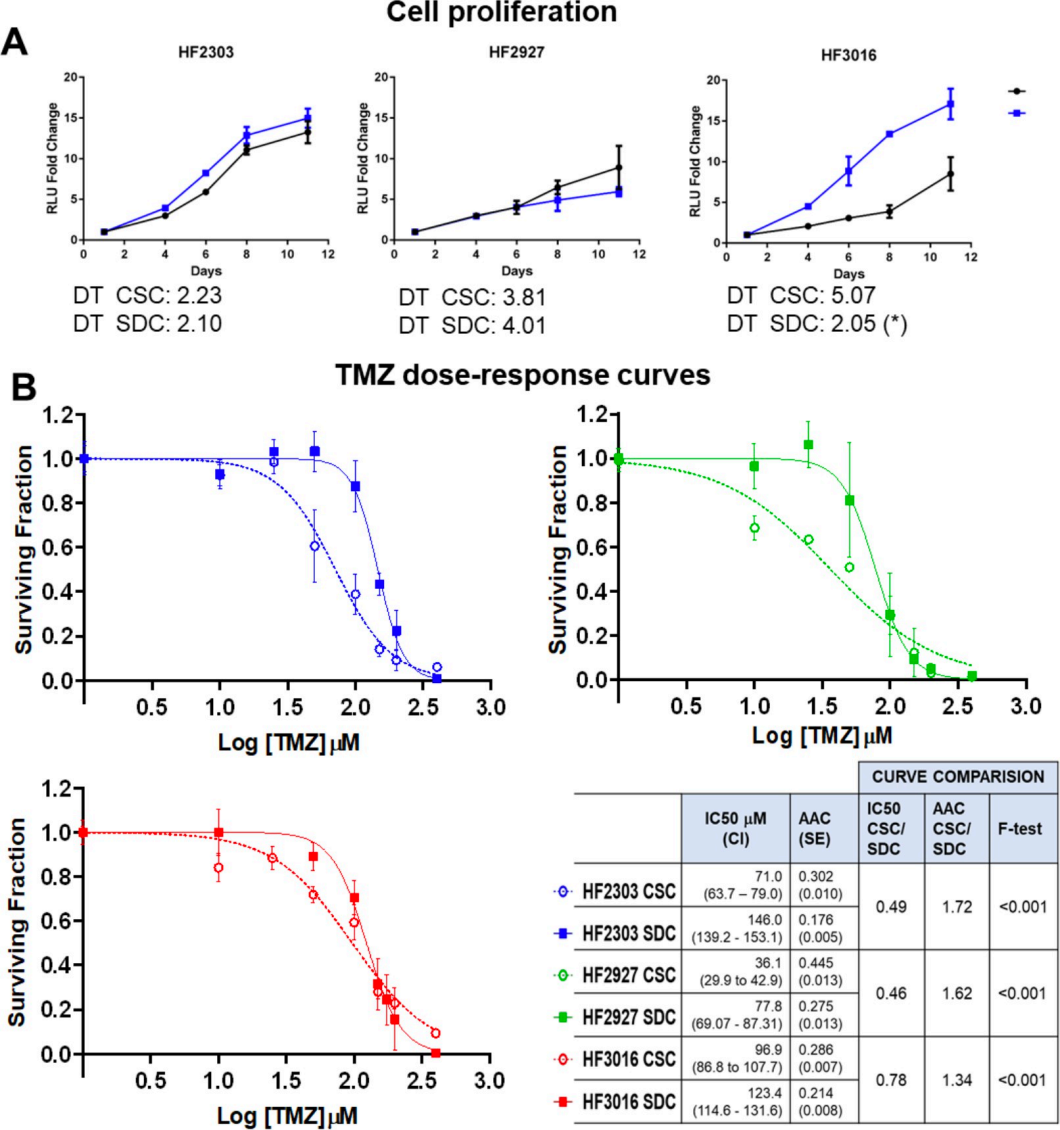
GBM CSCs are more sensitive to temozolomide than SDCs. **A)** To compare cell proliferation rates, CSCs and SDCs for each line were plated in the respective media in 96-well assay plates (1,000 cells/well) and cell viability was measured over 11 days in culture (n = 5). Graphs represent mean (SE). The calculated doubling-times (DT, days) are shown, pairwise comparison between CSC and SDC values were significant for HF3016 only (*) p<0.01. **B)** Cells were plated in 96-well assay plates (2,000/plate) and treated for 5 days with the indicated doses of temozolomide, or equivalent DMSO control. Dose-response curves, the calculated IC50 concentrations with confidence interval (CI), and area above the curve (AAC) expressed as a fraction of total area with SE are shown. For each of the GBM patients, CSCs were significantly more sensitive to TMZ relative to SDCs, as shown by IC50 and AAC ratios, and p-values for dose-response curve comparison (F-test).

MGMT epigenetic silencing is a clinically used biomarker to predict response to TMZ treatment. HF3016 and HF2303 CSCs were derived from tumors with unmethylated and HF2927 CSC from a tumor with methylated MGMT promoter (Fig 1A). Accordingly, HF2927 cells presented the lowest level of MGMT protein. Despite both having unmethylated promoter status, MGMT levels were 2.5-fold higher for HF3016 relative to HF2303 (Fig 4A, S1 Table). MGMT protein level in HF2303 was unchanged among all three culture conditions, while MGMT was significantly downregulated in SDCs relative to CSCs for HF2927 and HF3016 (Fig 4A). We confirmed that MGMT promoter was unmethylated for HF2303 and methylated for HF2927 cells and did not observe changes in methylation between CSC and SDCs for either of these models (S2 Fig). DNA methylation data is unavailable for HF3016 SDCs, preventing us from entirely ruling out differentiation-mediated alterations in MGMT promoter methylation in this model. We observed that MGMT protein expression pattern in the two models with unmethylated promoter, HF3016 and HF2303 (Fig 4A), was highly correlated (Spearman r = 0.823; 95% CI: 0.6213 to 0.9228) with the levels of activated NF-κB p65 (phospho-S536) (Fig 3B, S1 Table). Remarkably, the correlation between activated NF-κB and MGMT levels was significant for all models, regardless of promoter methylation status, but stronger for those presenting unmethylated MGMT (Fig 4B). To investigate to what extent the developmental state affected the sensitivity of GBM cells to DNA-damaging treatments, HF2303, HF2927 and HF3016 CSCs, along with their SDC progenies, were treated with TMZ and RT (Fig 1C). These models represent diverse molecular characteristics known to modulate response to DNA-damaging agents, such as TP53 status and MGMT promoter methylation. First, we compared cell proliferation rates between 3D CSC and 2D SDC cultures. SDCs proliferated at a higher rate than CSCs for HF3016 only, while no significant difference was observed for the other two lines (Fig 5A). Cells were then treated for 4 days with doses ranging from 0 (DMSO) to 400 mM TMZ, in quintuplicates. To compensate for possible binding of TMZ by proteins in FBS, even at the low concentration used, the serum-free neurosphere media was supplemented with bovine serum albumin (1%) [50]. Half maximal inhibitory concentration (IC50) and area above the curve (AAC) were calculated from dose-response curves generated by non-linear fitting. HF2927 cells were more sensitive to TMZ in comparison with the other two cell lines (Fig 5B), as expected based on MGMT promoter methylation and expression (Fig 4A). Differentiation of CSCs into SDCs significantly increased resistance to TMZ for all three lines (Fig 5B), despite downregulation of MGMT protein observed for HF2927 and HF3016 SDCs (Fig 4A).

**Fig 6:**
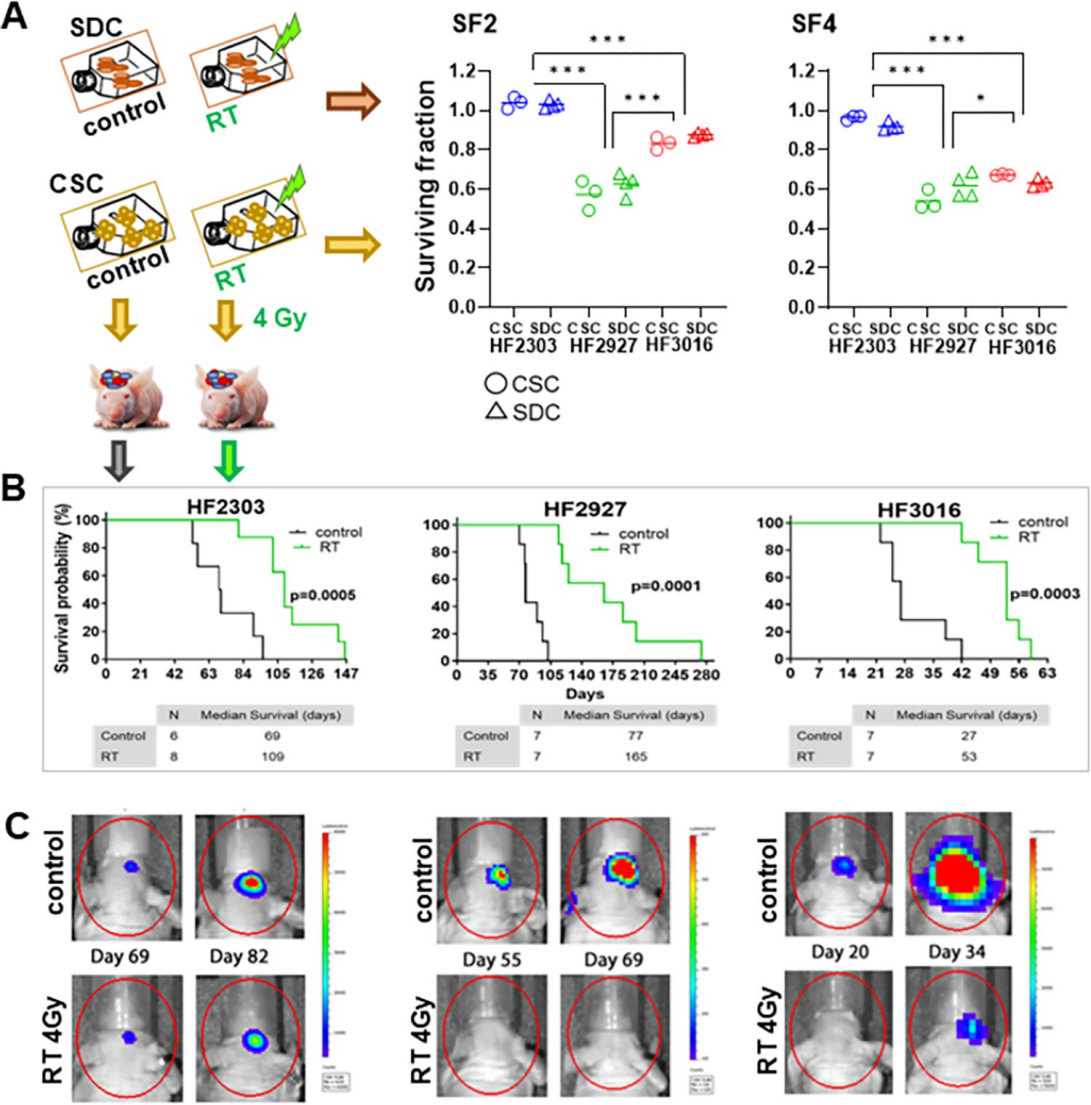
Response of glioblastoma CSCs and SDCs to ionizing radiation. **A)** CSC and SDC received a single radiation dose (2Gy or 4Gy) or mock radiation (control), and cell viability was measured 5 days later (n = 3–4). No significant difference in sensitivity to RT between CSC and SDCs was observed for any of the lines (Holm-Sidak multiple comparisons test with adj p-value<0.05 threshold). The surviving fraction (SF) values among the 3 cell lines (CSC and SDC combined) were compared by one-way ANOVA, followed by Tukey’s multiple comparisons tests, adj p value<0.0001(***), <0.05 (*). **B)** Irradiated and control CSCs were implanted intracranially immediately after treatment, 3x10^5^ cells/mouse (n = 6-8/group). Symptom-free survival for the orthotopic PDX is shown in Kaplan-Meier curves, compared by log-rank test. **C)** Longitudinal live bioluminescense images for representative PDX in (B).

We then compared the sensitivity of CSCs and SDCs to clinically relevant RT doses. Isogenic populations of CSCs and their SDC progenies were exposed to 2 gray (Gy) and 4 Gy RT doses and cultured for 5 days, surviving fractions (SF2 and SF4) relative to control treatment were determined (Fig 6A) as previously described [51]. Sensitivity to RT was variable among these models, as a high degree of resistance was observed for HF2303 cells relative to both HF3016 and HF2927; HF2927 was the most sensitive (Fig 6A). On the other hand, no significant difference in sensitivity to RT between CSCs and SDCs were observed for these three models (Fig 6A). To evaluate the long-term effect of radiation, we compared the tumorigenic potential of control and RT treated CSCs. Cells expressing firefly luciferase (fLuc) were implanted intracranially in nude mice immediately after radiation (4 Gy), or mock radiation (control). Tumor growth was monitored by weekly bioluminescence imaging and by daily monitoring of symptoms associated with tumor burden. The three irradiated CSC lines exhibited a delay in tumor growth relative to controls, assessed by symptom-free survival, with all mice eventually developing tumors, demonstrating that cells surviving 4 Gy RT dose were tumorigenic. HF2303 displayed the highest resistance to RT in vitro and exhibited the least significant difference in the survival curves. On the other hand, the HF2927 cells, which were the most sensitive to RT in vitro, displayed the lowest p-value in the curve comparison (Fig 6B and 6C). The degree of difference in survival curves (Log-rank p-values) ranked according to the in vitro sensitivity of each cell line to RT.

### Transcriptional reprogramming in response to CSC differentiation

**Fig 7:**
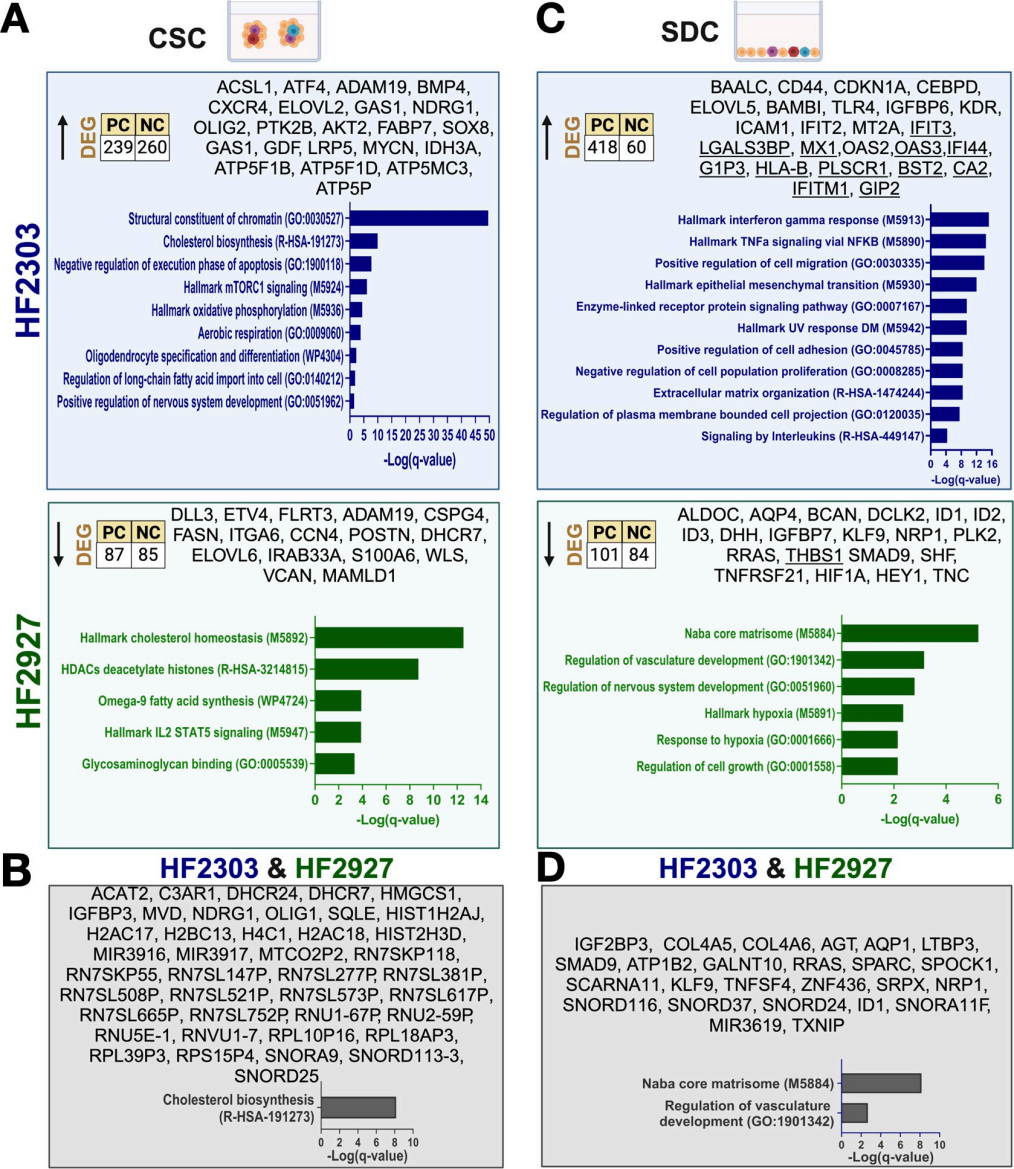
Transcriptional reprograming in glioblastoma cells in response to CSC differentiation. HF2927 and HF2303 CSC were grown in regular neurosphere media or in SDC cultures for 2 weeks. Total RNA from triplicate samples for each group was sequenced analyzed and DEG between CSC and SDC determined for each line. Total number of protein coding (PC) and non-coding (NC) genes, select genes and Metascape gene-set enrichment tests adjusted for multiple comparisons (q-value < 0.05) are shown for genes upregulated in CSCs (A) and in SDCs (C). The common DEGs for both lines along with enriched cellular processes are presented: 43 genes upregulated in CSCs (B) and 25 genes upregulated in SDCs (D). Figure generated using BioRender (Created in BioRender. Berezovsky, A. (2024) https://BioRender.com/n49q520).

To determine how differentiation state impacts transcriptional reprogramming in response to TMZ and RT, we selected the most resistant (HF2303) and the most sensitive (HF2927) models. We first determined the pattern of global transcriptome changes after differentiation of CSCs into SDCs from bulk RNAseq data. To identify differentially expressed genes between the two differentiation states, we employed the non-parametric NOISeq R package, and subsequently performed pathway enrichment analysis, as detailed in the Materials and Methods section. Numerous signaling pathways and cellular processes enriched in CSCs were cell line specific. It has been proposed that CSCs, from GBMs and other cancers, tend to rely more on oxidative phosphorylation (OXPHOS) than the more differentiated cancer cells [52]. Here, we observed that OXPHOS gene expression signature was enriched in HF2303 CSCs but not HF2927 CSCs (Fig 7A). Interestingly, reserve respiratory capacity was higher in untreated HF2303 relative to HF2927 cells, and higher in SDC compared to CSCs (S3 Fig). Replication-dependent histones were highly enriched in HF2303 CSCs, in the absence of enrichment in cell proliferation markers (Fig 7A, S2 Table), as previously observed for embryonic and induced pluripotent stem cells [53]. Several nuclear pseudogenes with sequence similarity to mitochondrially encoded 16S rRNA (e.g. *MTRNR2L1*, *MTRNR2L3*), enriched in HF2303 CSCs (A in S2 Table) are predicted to encode humanin-like peptides and to be involved in negative regulation of the execution phase of apoptosis [54] (Fig 7A). IL2-STAT5 signaling was specifically upregulated in HF2927 CSC in relation to SDC (Fig 7A), in agreement with levels of pSTAT5_Y694 being elevated in HF2927 CSC (4.697 ± 0.98), while undetectable in HF2927 SDCs and HF2303 cells (B in S1 Table). Patterns of transcriptome reprogramming in response to differentiation that were common to both HF2927 and HF2303 models included downregulation of genes involved in cholesterol biosynthesis and upregulation of genes associated with ECM components and organization, including collagen type IV alpha 6 chain (*COL4A6*), a target of SRY-box transcription factor 2 (*SOX2*) transcriptional regulation [55] (Fig 7B). The expression of several transcription factors associated with stemness and pluripotency, such as *SOX2*, spalt like transcription factor 2 (*SALL2*) and POU class 5 homeobox 1 (*POU5F1* (Oct3/4)), was not altered upon differentiation of HF2303 and HF2927 CSCs into SDCs (A,B in S2 Table). On the other hand, other neural progenitor and stemness markers expressed in CSCs were down regulated upon differentiation: oligodendrocyte transcription factor 1 (*OLIG1*) in both lines; delta like canonical Notch ligand 3 (*DLL3*), integrin subunit alpha 6 (I*TGA6*), ETS variant transcription factor 4 (*ETV4*) in HF2927; fatty acid binding protein 7 (*FABP7*), oligodendrocyte transcription factor 2 (*OLIG2*) and ELOVL fatty acid elongase 2 (*ELOVL2*) [56] in HF2303 (Fig 7A and 7B).

IFN and interleukins signaling were specifically upregulated in HF2303 SDCs (Fig 7C). Indeed 29 genes associated with IFN response pathways were upregulated in HF2303 SDCs (Table 1), including members of the 49-gene interferon-related DNA damage resistance signature (IRDS) [57]. Differentiation of CSCs into SDCs resulted in enrichment of the epithelial mesenchymal transition (EMT) signature for HF2303 but not for HF2927 (Fig 7C), consistent with the transcriptional subclasses affiliation, mesenchymal for HF2303 and classical for HF2927 (Fig 1A). In agreement with our observation that receptor dependent SMAD1/3/8 are activated in GBM SDCs (Fig 2A), inhibitor of DNA binding 1 (*ID1*), *ID2* and *ID3*, BMP/retinoic acid inducible neural specific 1 (*BRINP1*), downstream of BMP signaling, were upregulated in HF2927 SDCs (Fig 7C). Several components of the TGFb superfamily signaling were upregulated in HF2303 SDCs, including transforming growth factor beta receptor 2 (*TGFBR2*), *BMP1*, latent transforming growth factor beta binding protein 1 (*LTBP1*), *LTBP3*, *SMAD3*, *SMAD9* (Fig 7C and 7D; S2 Table). On the other hand, endogenous *BMP4* mRNA was downregulated and BMP antagonists DAN family BMP antagonist (*NBL1*) and BMP and activin membrane bound inhibitor (*BAMBI*) were upregulated in HF2303 SDCs, suggesting a possible negative feedback loop, specific for this model. BAALC binder of MAP3K1 and KLF4 (*BAALC)*, a regulator of developing neuroectoderm tissues overexpressed in acute leukemia and GBM [58], and a target of SOX2 in differentiated GBM cells [26], was upregulated in HF2303 SDCs (Fig 7C). Toll like receptor 4 (*TLR4*), which is expressed in both immune and neoplastic cells, was also upregulated in HF2303 SDCs (Fig 7C), in agreement with previous studies [59]. *HIF1A*, *an* IFN responsive gene (Table 1), along with downstream hypoxia markers were uniquely upregulated in HF2927 SDCs, under normal oxygen levels (Fig 7C)`. The 24 genes commonly upregulated in SDCs for both lines were enriched in ECM components (Fig 7D). Small nucleolar RNAs were also upregulated upon differentiation of both HF2303 and HF2927 (Fig 7D; S2 Table), including SNORD116 cluster, shown to have non-canonical functions in the differentiation, survival, and proliferation of neuronal cells [60].

**Table 1:** Differentially expressed genes associated with IFN response and signatures.

Gene Symbol	DEG in response to 2% FBS differentiation		DEG in response to TMZ		DEG in response to RT		IFN response
	Upregulated	Downregulated	Upregulated	Downregulated	Upregulated	Downregulated	
***ACSL5***	-	-	HF2927 SDC	-	-	-	a
***ODF3B***	-	-	HF2927 SDC	-	-	-	a
***NEAT1***	-	-	HF2927 SDC	-	-	-	d
***SECTM1***	-	-	HF2927 SDC	-	-	-	c
***OASL***	-	-	HF2927 SDC; HF2927 CSC	-	-	-	a, b, c, d
***CDKN1A***	HF2303	-	HF2927 SDC; HF2927 CSC	-	-	-	c
***RSAD2***	-	-	HF2927 SDC; HF2303 SDC	-	-	-	a, b, c
***CMPK2***	-	-	HF2927 CSC	-	-	-	a, b, c
***CXCL10***	-	-	HF2927 CSC	-	-	-	a, b, c, d
***EPSTI1***	-	-	HF2927 CSC	-	-	-	a, b, c
***DHX58***	-	-	HF2927 CSC	-	-	-	a, b, c
***MX2***	-	-	HF2927 CSC	-	-	-	a, c, d
***ISG15***	HF2303	-	HF2927 CSC	-	-	-	a, b, c, d
***TRIM22***	-	-	HF2927 SDC; HF2927 CSC	-	HF2927 CSC	-	a
***MX1***	HF2303	-	HF2927 CSC; HF2303 CSC; HF2303 SDC	-	HF2927 CSC	-	a, b, c, d, e
***IFI6***	HF2303	-	HF2927 CSC	-	HF2927 CSC	-	a, d
***OAS2***	-	-	-	-	HF2927 CSC	-	a, c
***HERC6***	-	-	-	-	HF2927 CSC	-	a, b, c, d
***IFI44L***	-	-	-	-	HF2927 CSC	-	a, b, c, d
***HLA-E***	HF2303	-	-	-	HF2927 CSC	-	a
***OAS1***	-	-	HF2303 SDC	-	-	-	a, b, d, e
***OAS3***	-	-	HF2303 SDC	-	-	-	a, c, d
***SAMD9L***	-	-	HF2303 SDC	-	-	-	a, b, c
***GIMAP2***	-	-	-	HF2927 SDC	-	-	a
***TNFAIP3***	-	-	-	HF2927 SDC	-	-	c
***THBS1***	-	HF2927	-	HF2927 SDC	-	-	d
***TNFAIP6***	-	HF2927	-	HF2927 SDC	-	-	c
***HLA-B***	HF2303	-	-	-	-	HF2303 CSC	a, c, d
***IGF2***	-	-	-	-	-	HF2303 CSC	d
***MTHFD2***	-	HF2303	-	-	-	-	c
***IFIT3***	HF2303	-	-	-	-	-	a, b, c, d, e
***BST2***	HF2303	-	-	-	-	-	a, b, c, d
***IFI44***	HF2303	-	-	-	-	-	a, b, c, d, e
***RIGI***	HF2303	-	-	-	-	-	a, c
***IRF9***	HF2303	-	-	-	-	-	a, b, c
***IFIT2***	HF2303	-	-	-	-	-	a, b, c
***IFITM1***	HF2303	-	-	-	-	-	a. b. d
***PLSCR1***	HF2303	-	-	-	-	-	a, b, c, d
***PLAAT4***	HF2303	-	-	-	-	-	a
***HLA-C***	HF2303	-	-	-	-	-	a, b
***LGALS3BP***	HF2303	-	-	-	-	-	a, b, c, d
***CA2***	HF2303	-	-	-	-	-	d
***ARL4A***	HF2303	-	-	-	-	-	c
***BTG1***	HF2303	-	-	-	-	-	c
***C1R***	HF2303	-	-	-	-	-	c
***CCL2***	HF2303	-	-	-	-	-	c
***FGL2***	HF2303	-	-	-	-	-	c
***ICAM1***	HF2303	-	-	-	-	-	c
***MVP***	HF2303	-	-	-	-	-	c
***NAMPT***	HF2303	-	-	-	-	-	c
***PELI1***	HF2303	-	-	-	-	-	c
***UPP1***	HF2303	-	-	-	-	-	c
***MT2A***	HF2303	HF2927	-	-	-	-	c
***HIF1A***	HF2927	-	-	-	-	-	c

(a) S3 Table TCGA IFN response correlation group; (b) M5911 Hallmark IFN alpha response https://www.gsea-msigdb.org/; (c) M5913 Hallmark IFN gamma response https://www.gsea-msigdb.org/; (d) IRDS ref. [57]; (e) IFN/STAT1 survival proneural ref. [63].

### Transcriptional response to sublethal treatment with temozolomide and radiation is determined by genomics and differentiation state

**Fig 8:**
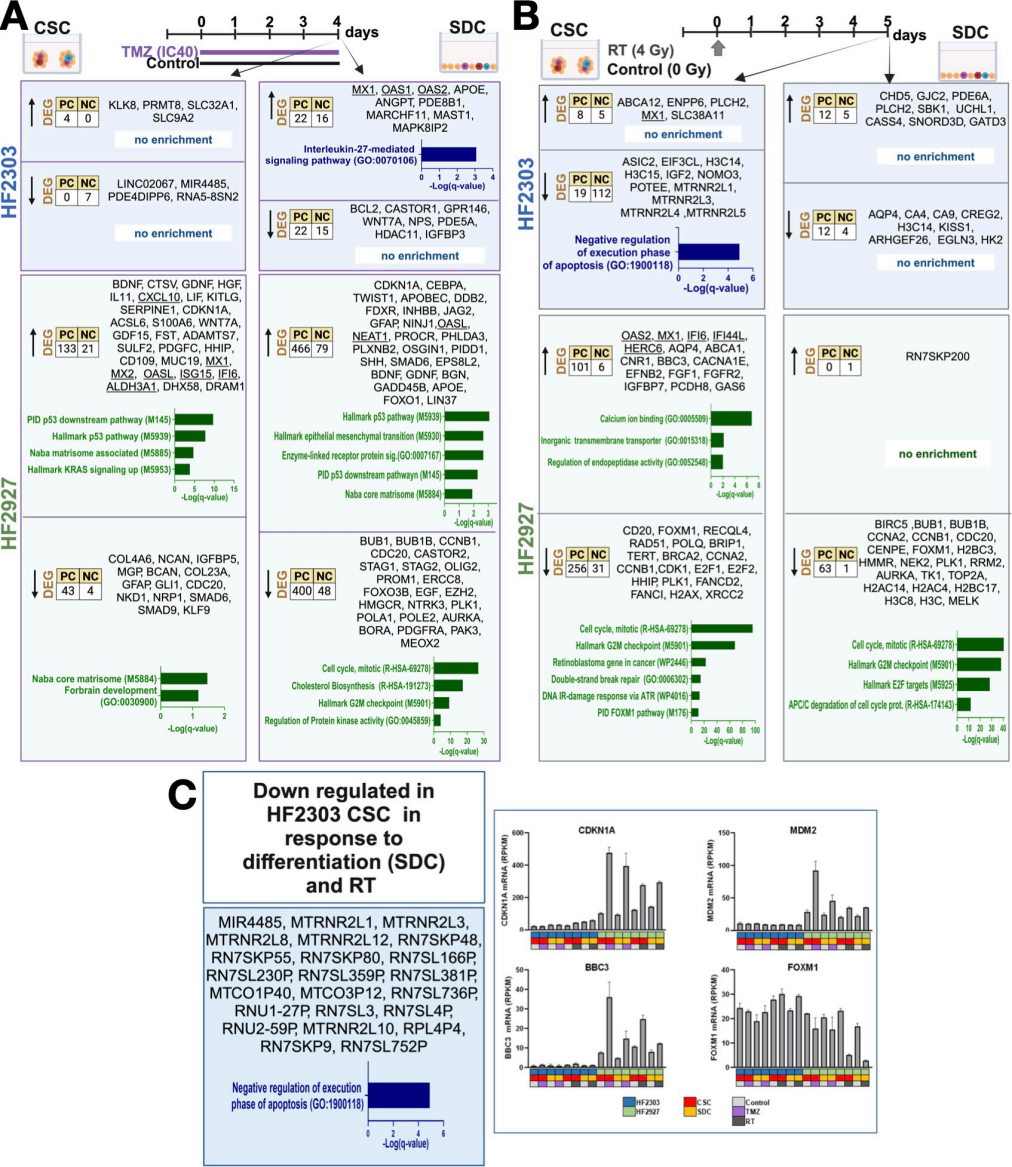
Transcriptional response to genotoxic treatment in glioblastoma cells. Differentially expressed genes between treated and control samples were determined and enrichment performed as described for Fig 7. **A)** Transcriptional changes in CSCs and SDCs in response to 4-day TMZ treatment (IC40 concentrations). **B)** Transcriptional changes in CSCs and SDCs measured 5 days after one dose RT (4 Gy). **C)** RNA commonly downregulated in HF2303 CSCs in response to differentiation and RT treatment. **D)** mRNA expression (RPKM) of p53 transcriptional targets CDKN1A (p21), MDM2, and BBC3, and of cell cycle regulators in HF2303 and HF2927 control and treated cells, mean and SE of n = 3. Figure created with Biorender.com (Created in BioRender. Berezovsky, A. (2024) https://BioRender.com/n49q520).

To compare the transcriptional response to temozolomide treatment at distinct differentiation states, GBM cells were treated in triplicate with the respective TMZ IC40 concentrations, calculated from dose-response curves in Fig 5B: 58 mM (HF2303 CSC), 25 mM (HF2927 CSC), 134 mM (HF2303 SDC), 70 mM (HF2927 SDC), or equivalent DMSO control for 4 days. Cells were then harvested and total RNA isolated and sequenced. Differentially expressed genes between treated and control samples for each model and culture type were determined as described for Fig 7 (C and D in S2 Table). Transcriptional response to 4-day TMZ treatment was largely regulated by wt p53 activation in HF2927 cells (Fig 8A). We observed that 18 out of the 36 genes commonly upregulated in HF2927 CSCs and SDCs in response to TMZ treatment (Table 2) are direct transcriptional targets of p53 [55]. However, the differentiation state of HF2927 cells also influenced p53 transcriptional specificity, as 23 and 26 p53 target genes were significantly upregulated in response to TMZ exclusively in CSCs or SDCs, respectively (Table 2). In contrast, interleukin-27 (IL-27) mediated signaling pathway was enriched in HF2303 SDC in response to TMZ (Fig 8A), based on the upregulation of Myxovirus resistance dynamin like GTPase 1 (*MX1*), 2’ 5’ oligoadenylate synthetase 1 (*OAS1*) and *OAS2*, all of which are members of the IRDS [57], providing further evidence for the proposed cooperation between IL-27 and IFN signaling in response to DNA damage [61]. To identify other components of IFN signaling in GBMs, we identified 100 genes whose expression presented the highest correlation to *OAS1* (Spearman’s r > 0.55; q value < 5.0E-12) in The Cancer Genome Atlas (TCGA) GBM data set (CBIOPortal [62], accessed on 01/19/24). 62% of these genes are members of the hallmark IFN alpha or gamma response pathways, 24% are members of the IRDS [57], and this gene list also includes the IFN/STAT1 prognostic signature proposed for GBM proneural subtype [63] (S3 Table). We observed activation of IFN response in TMZ-treated HF2927 CSCs and SDCs, and in HF2303 SDCs (Fig 8A, Table 1). TMZ treatment induced expression of ECM components, and several neural developmental growth and survival factors, including brain derived neurotrophic factor (*BDNF*) and glial cell derived neurotrophic factor (*GDNF*), in HF2927 CSCs and SDCs (Fig 8A). Expression of other genes associated with neural development were down regulated in TMZ treated HF2927: platelet derived growth factor receptor alpha (*PDGFRA*), *OLIG2*, and prominin 1 (*PROM1*) in SDCs; neuropilin 1 (*NRP1*), GLI family zinc finger 1 (*GLI1*), and Kruppel like factor 9 (*KLF9*) in CSCs (Fig 8A). *CDKN1A* (p21), the main effector of p53-mediated downregulation of cell cycle genes, leading to cell cycle arrest, was upregulated in response to TMZ in both HF2927 CSCs and SDCs (Fig 8A and 8D). The more pronounced downregulation of cell cycle genes in HF2927 SDCs relative to CSCs (Fig 8A) is consistent with corresponding upregulation of Lin-37 DREAM MuvB core complex component (*LIN37*) in SDC but not in CSC (Fig 8A), as LIN37 is required for transcriptional repression by the DREAM complex, downstream of p53 signaling [64]. A possible explanation for these observations is that cell cycle-arrested TMZ-treated HF2927 CSCs may have undergone apoptosis to a greater extent at the time point analyzed, as they demonstrated increased sensitivity to TMZ treatment (Fig 5B). Furthermore, genes coding for proteins involved in EMT, including the master regulator twist family bHLH transcription factor 1 (*TWIST1*), were upregulated in response to TMZ exclusively in HF2927 SDCs, which could contribute to the increased resistance of the differentiated cells.

**Table 2:** Genes upregulated in HF2927 cells in response to temozolomide.

Validated p53 direct targets			Other
CSC & SDC	CSC	SDC	CSC & SDC
*ABCA12*	*ALDH1A3*	*APOBEC3C*	*ABCA1*
*ACER2*	*ASTN2*	*CATSPERG*	*ANK1*
*BBC3*	*CEL*	*CES2*	*APLP1*
*CDKN1A*	*CLCA2*	*CGB7*	*BDNF*
*COL7A1*	*DRAM1*	*DDB2*	*CARNS1*
*CPE*	*EDA2R*	*EML2*	*F11R*
*DGKA*	*FAM13C*	*GPX1*	*FOXI3*
*EPS8L2*	*HES2*	*IGFBP7*	*FST*
*FDXR*	*KLHDC7A*	*MR1*	*GALNT5*
*GDF15*	*LAPTM5*	*MYOF*	*GDNF*
*KITLG*	*LIF*	*NINJ1*	*HKDC1*
*PLK3*	*MAST4*	*NKAIN4*	*INPP5D*
*SERPINE1*	*MDM2*	*PARD6G*	*IP6K3*
*SPATA18*	*NEFL*	*PGF*	*KIAA1217*
*SULF2*	*PDE4C*	*PHLDA3*	*KISS1R*
*TP53I3*	*PLCL2*	*PIDD1*	*LYNX1*
*TRIM22*	*PRDM1*	*PLXNB2*	*MARCH11*
*WDR63*	*RGS16*	*PRKX*	*MUC19*
	*RRM2B*	*RGMA*	*NECTIN4*
	*SESN1*	*RRAD*	*NEURL1*
	*SYTL1*	*SCN2A*	*NR5A1*
	*TP53INP1*	*SCN4B*	*NTN1*
	*VWCE*	*SESN2*	*OASL*
		*TNFRSF10C*	*PDGFC*
		*TSPAN11*	*PROCR*
		*UNC5B*	*SLC52A1*
			*SMIM10L2A*
			*STC2*
			*TMEM80*

Ionizing radiation can damage molecules directly, exerting most of its toxicity to cancer cells through DNA double strand breaks (DSBs), and indirectly through the generation of free radicals. The complex cellular responses leading to cell cycle arrest, apoptosis, DNA repair and other processes occur over time [65]. The relative sensitivity of glioblastoma cells to RT varied according to tumor of origin but not according to differentiation status under the experimental parameters employed here (Fig 6A). Furthermore, pre-implantation treatment of CSCs with 4Gy led to delayed xenograft tumor growth for all models (Fig 6B). To integrate these observations with cellular responses, CSCs and SDCs were treated in triplicates with 4 Gy radiation dose or mock radiation control, followed by incubation in the respective growth media for 5 days prior to RNA isolation and sequencing. Under these conditions, few transcripts were altered in response to RT in HF2303 (Fig 8B), consistent with the resistance to 4 Gy dose observed for both SDCs and CSCs (Fig 6A). The only transcripts commonly upregulated in HF2303 CSCs and SDCs in response to RT were phospholipase C eta 2 (*PLCH2*) and *SNORD3D* (Fig 8B). Interestingly, 50 of the pseudogenes involved in negative regulation of the execution phase of apoptosis, which were downregulated in response to differentiation of CSCs into SDCs (Fig 7), were also downregulated in RT-treated HF2303 CSCs (Fig 8B and 8C). Compared to TMZ treatment, fewer IFN response genes were upregulated in RT treated cells, including *MX1* which was upregulated in both CSC lines, and *OAS2*, *HERC6*, *IFI44L* and *HLA-E* in HF2927 CSCs (Table 1, Fig 8B). In contrast to the response to TMZ, where the cells were analyzed after 4 days of continuous treatment (Fig 8A), p53-mediated signaling in HF2927 was more attenuated 5 days after RT, as expected (Fig 8B). Few p53 transcriptional targets remained elevated in irradiated HF2927 cells, such as BCL2 Binding Component 3 (*BBC3 / PUMA*), a pro-apoptotic BCL-2 family member, while expression of several others, including *MDM2*, were not altered at the 5-day timepoint (Fig 8B and 8D). *CDKN1A*/p21, the main effector of p53 mediated cell cycle arrest significantly upregulated in response to TMZ (Fig 8A), was elevated to a lesser degree in response to RT, below the statistical threshold employed to determine DEGs (Fig 8D). However, sustained downregulation of cell cycle genes downstream of p53 activation was observed for RT-treated HF2927 CSCs and SDCs (Fig 8B). Indeed, 53 out of the 56 transcripts commonly downregulated in HF2927 CSC and SDCs in response to RT are transcriptional targets of the DREAM (dimerization partner, RB-like, E2F and multivulval class B) repressive complex [66] (Table 3). Among these targets, forkhead box M1 (*FOXM1*), a transcriptional activator of cell cycle genes, especially those involved in G2 and M phases [67], along with its transcriptional targets, including Polo-like kinase 1 (*PLK1*) which phosphorylates and activates FOXM1 in a positive feed-back loop [68], were downregulated exclusively in RT-treated cells at the analyzed time point (Fig 8B and 8D). Furthermore, DNA damage response and double strand break repair genes were downregulated in response to RT predominantly in HF2927 CSCs (Fig 8B), indicating that these cells would be sensitized to subsequent DNA damaging interventions.

**Table 3:** Genes downregulated in HF2927 CSC and SDC in response to radiation.

RT CSC/SDC	RT CSC/SDC & TMZ SDC	RT CSC/SDC & TMZ CSC/SDC
AURKB (*)	ASPM (*)	CDC20 (*)
BIRC5 (*)	AURKA (*)	KIF20A (*)
CCNA2 (*)	BUB1 (*)	
CCNB2 (*)	BUB1B (*)	
CDCA2 (*)	CCNB1 (*)	
DEPDC1 (*)	CDCA8 (*)	
ESPL1 (*)	CENPE (*)	
FOXM1 (*)	CEP55 (*)	
GTSE1 (*)	DLGAP5 (*)	
HIST1H1B (*)	GAS2L3 (*)	
HJURP (*)	HIST1H2AB (*)	
HMGB2 (*)	HIST1H2AJ (*)	
HMMR (*)	HIST1H2AL (*)	
IQGAP3 (*)	*HIST1H2BB*	
KIF15 (*)	HIST1H2BO (*)	
KIF23 (*)	HIST1H3F (*)	
KIF2C (*)	HIST1H3G (*)	
MELK (*)	KIF11 (*)	
NEK2 (*)	KIF14 (*)	
NUSAP1 (*)	KIFC1 (*)	
*PIMREG*	*KNL1*	
PRC1 (*)	MKI67 (*)	
PTTG1 (*)	NCAPH (*)	
RRM2 (*)	NUF2 (*)	
TROAP (*)	PBK (*)	
UBE2C (*)	PLK1 (*)	
	TPX2 (*)	
	TTK (*)	

(*)Transcriptional target of the DREAM repressor complex.

### DNA methylation alteration pattern in differentiation of CSCs

**Fig 9:**
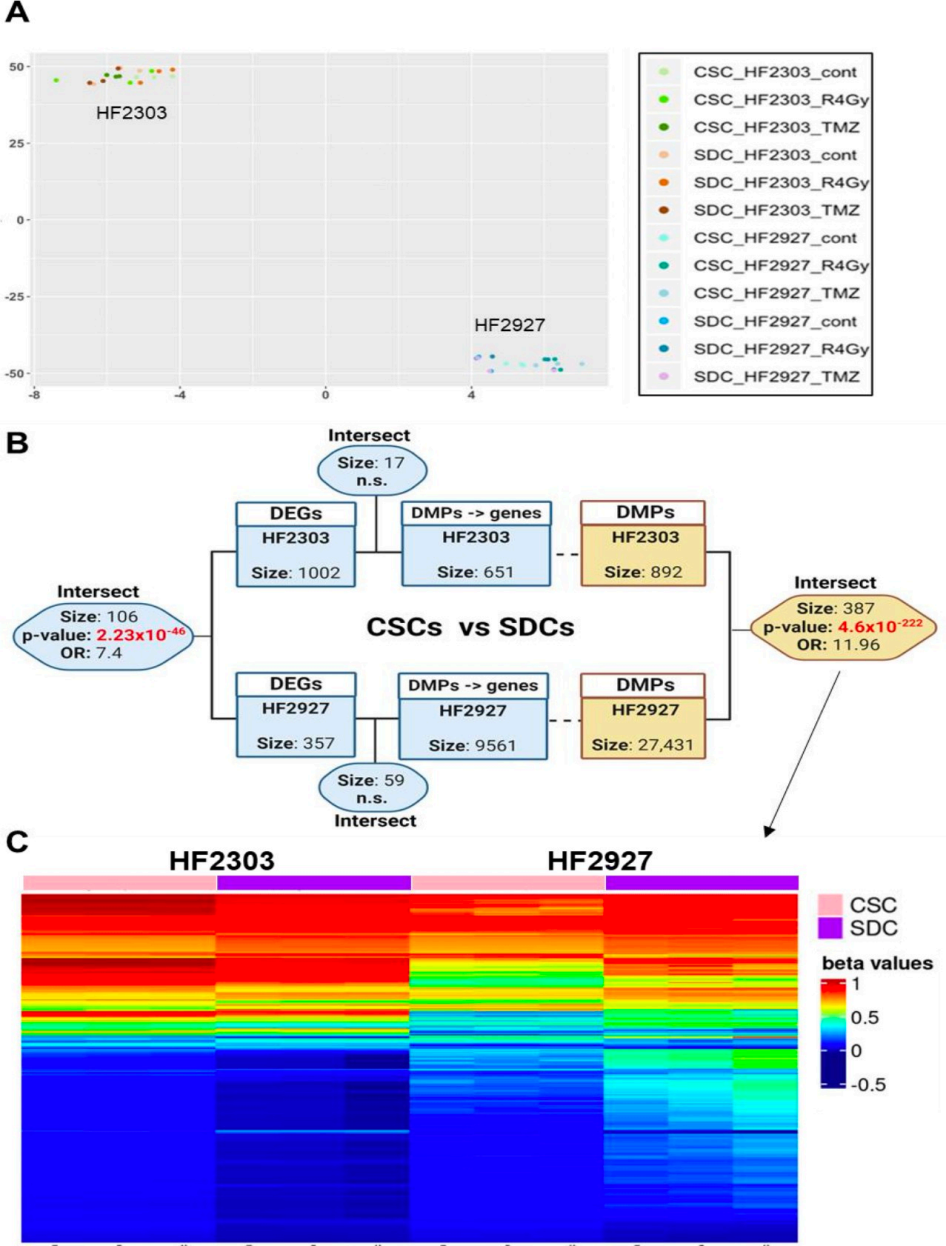
Genome-wide DNA methylation profiling groups samples by patient of origin and identifies DNA methylation alteration patterns between glioblastoma CSCs and their SDCs progeny. **A)** t-distributed stochastic neighbor embedding (t-SNE) plot from β-values for all CpG sites comparing HF2303 and HF2927 CSCs and SDCs treated with control, RT or TMZ, in triplicates. **B)** Schematic showing the significant intersect for HF2303 and HF2927 of differentially expressed genes (DEGs) and differentially methylated positions (DMPs) between CSCs and SDCs. The intersect between DMPs mapped to genes (see Methods) and DEGs are shown. Significance of the intersections was determined using GeneOverlap, p-values and estimated odds ratio are shown; n.s., nonsignificant. **C)** Heatmap showing the β-values for the 387 DMPs common to HF2303 and HF2927. Fig 9B was created with Biorender.com (Created in BioRender. Berezovsky, A. (2024) https://BioRender.com/n49q520).

To verify the contribution of epigenetic regulation to the transcriptional responses reported above, we analyzed the global DNA methylation profile of control and treated HF2303 and HF2927 CSCs and SDCs in triplicates, using Infinium Human Methylation 450K BeadChip. Differences in DNA methylation distinguished the samples according to the tumor of origin (HF2303 vs HF2927), as shown in the t-distributed stochastic neighbor embedding (t-SNE) plot (Fig 9A). Analysis of the differentially methylated positions (DMPs) and regions (DMRs) between CSC and SDC for each of these models identified 892 DMP and 12 DMRs for HF2303, and 27,431 DMP and 2 DMRs for HF2927 (S4 Table). Similar to the significant overlap in DEGs associated with CSC differentiation between the two cell lines (Figs 7B, 7D and 9B), we identified 384 DMPs common to both lines (Fig 9B, S4 Table), with a pattern of decreased b-values in SDCs relative to CSCs in HF2303, while HF2927 SDCs exhibited an increase in b-values. The intersection between genes mapped to the DMPs, at the transcription start sites or within CpG islands, and the genes differentially expressed between CSCs and SDCs were limited to 17 and 59 genes for HF2303 and HF2927, respectively. Among these genes, latent transforming growth factor beta binding protein 3 (*LTBP3*) was associated with DMPs and upregulation in SDC for both models (Fig 9B, S4 Table). Cholesterol homeostasis, hypoxia, growth factor binding, and ECM components were significantly represented in the 59 gene list from HF2927 (S4 Table). No significant DNA methylation changes were observed in the acute response to RT and TMZ treatment across both models and differentiation states (S4 Fig).

## Discussion

Understanding the extent to which oncogenic signaling and biological pathways modulating CSC phenotype and differentiation are influenced by diverse genomic abnormalities, or are conversely generalizable to all GBMs, is crucial for the design and interpretation of biological and experimental therapeutical studies. For example, astrocytic differentiation of CSCs into SDCs resulted in the activation of RTK and MAPK pathways in most GBM cells, consistent with a role for MAPK in early astrocytic differentiation [69]. However, this activation was not observed for the two models carrying ecDNA EGFR amplification (HF2927 and HF3016). On the other hand, BMP-Smad pathway was prominently activated in all GBM models upon exposure to 2% and 10% FBS. Activation of BMP-Smad was further supported by the observation that transcriptional targets of r-Smad1/5/8 were upregulated in HF2303 and HF2927 SDCs. This is consistent with the use of BMP4 to induce the differentiation of iPSC into astrocytes [30], with BMP signaling inducing astrocytic differentiation of patient-derived oligodendroglioma cells [70], and with BMP ligands being present in FBS [33]. We found that signaling promoting cell survival in untreated GBM cells varies among the different models but not as much between CSCs and SDCs. For example, hyperactivation of the PI3K/AKT/mTOR pathway, which promotes cell growth, proliferation and survival, frequently occurring in GBMs [71], was present in both CSCs and SDCs for 7/8 GBM models. The integrated analysis of targeted proteomics in multiple GBM models, distinct differentiation states and treatment groups showed a complex co-activation and cross talk among oncogenic signaling pathways, which greatly contributes to aberrant cancer cell growth, proliferation, survival, and resistance to therapeutic interventions [24]. We show that culturing CSCs in media supplemented with 10% FBS resulted in suppression of key oncogenic signaling in all 8 GBM models, providing further evidence that these culture conditions are not conducive to preserving GBM molecular characteristics, as previously reported [28,47].

Earlier studies characterized GBM “CSCs” as a subpopulation of cells isolated from dissociated tumors based on expression of certain markers (e.g., CD133), which were subsequently cultured in neural stem cell media, while marker-negative cells were considered non-CSCs, or “differentiated”, and cultured in 10% FBS. Using this paradigm, these earlier studies attributed GBM marker positive “CSCs” with increased DNA repair capacity and corresponding resistance to RT, when compared to marker negative “non-CSCs”, e.g. [72]. In contrast, here GBM CSC subpopulations are propagated through enrichment in selective neurosphere media, a widely employed strategy [11,20,21], and differentiated GBM cells are defined as the progeny from astrocytic differentiation of CSCs in 2% FBS (SDCs). In this context, we report that sensitivity to clinically relevant doses of RT was unchanged between CSCs and their differentiated SDCs progeny, while SDCs presented increased resistance to TMZ for all three models tested, two representing unmethylated and one methylated MGMT promoter. We show that baseline MGMT protein levels are strongly correlated with NF-κB activation in GBM cells, independent of MGMT promoter methylation status. This finding aligns with previous studies indicating that NF-κB activation confers resistance to DNA-alkylating agents partially through regulation of MGMT expression in glioma cells [7]. NF-κB activation in response to DNA-damage, including that induced by TMZ treatment, has been previously reported [73]. Together, these findings underscore the potential of targeting NF-κB as an adjunct therapy to enhance TMZ efficacy in GBM, consistent with results in established GBM cell lines.[74]. However, clinical trials testing proteasome inhibitors, which prevent inhibitor of κB (IκB) degradation, in combination with TMZ in GBM have not achieved sufficient efficacy [75]. Selinexor (KPT-330), a selective small molecule inhibitor of nuclear export predicted to suppress NF-κB activity [76], is currently in clinical trials for GBM in combination with TMZ and RT (e.g. NCT04216329). Realizing the therapeutic potential of NF-κB inhibition in combination with DNA-damaging treatments for GBM will require the development of brain-penetrant inhibitors that specifically and potently target pro-survival NF-κB signaling pathways.

Transcriptional reprograming associated with the differentiation of CSCs into SDCs revealed two commonly altered cellular functions in the GBM models analyzed. Cholesterol biosynthesis was enriched in CSCs, with evidence for transcriptional activity of sterol regulatory element binding proteins (SREBPs), in response to the lack of cholesterol in the serum-free neurosphere media. Because GBM cells tend to uptake cholesterol from the brain microenvironment [77] and cholesterol is a component of FBS, cholesterol biosynthesis was downregulated in SDCs (2% FBS). ECM components, receptors and regulators were upregulated in SDCs, resembling transcriptional regulation of ECM components in astrocytes [78]. The enrichment in ECM-receptor interaction observed for SDCs is associated with treatment resistance GBMs [79], and could be a factor contributing to the increased resistance of GBM SDCs to TMZ treatment. The retention of the expression of key stemness signature genes in SDCs, including *SOX2* and several of its transcriptional targets, highlights the role of these genes in the maintenance of developmental plasticity in more differentiated GBM cells, as we have previously reported [26]. Other aspects of the transcriptional reprograming associated with differentiation of CSCs were model specific, such as the enrichment of EMT signature specifically in differentiated CSC from a GBM morphological variant gliosarcoma [26]. We observed common DNA methylation alterations associated with CSC differentiation between the 2 lines. These findings parallel the reverse epigenetic modifications in reprogramming SDCs to CSCs [15] and suggest that differentiation cues converge on common epigenetic profiles in GBM samples. Here, we did not observe significant acute alterations in global DNA methylation patterns in response to RT or TMZ treatment, in line with the variability in the occurrence and extent of DNA methylations changes in response to treatment cultured cells [80].

We observed that both genomics and cell developmental states impact GBM response to TMZ and RT. The status of *TP53*, a key regulator of cellular response to DNA-damaging agents, was not surprisingly a key determinant of the transcriptional response of the GBM models to TMZ and RT. HF2927 carries wt TP53 while HF2303 carries TP53 G245S, a dominant negative mutation which disrupts the structure of p53 DNA binding domain, impairing p53 transcriptional activity [81]. Furthermore, wt p53 transcriptional activation was modulated by differentiation status, most notably in TMZ treated cells. Downregulation of cell cycle genes by p53 in response to DNA-damage, accomplished indirectly through the DREAM repressive complex [66], was observed for wt p53 CSCs and SDCs in response to TMZ and RT. *FOXM1*, a transcriptional activator of cell cycle genes overexpressed in various cancers [67], and a DREAM target [66], was highly expressed in untreated HF2303 and HF2927 cells and downregulated exclusively in HF2927 CSCs and SDCs 5 days after RT, indicating the prolonged effect of cell cycle arrest through downregulation of cell cycle genes, downstream of p53 activation. These results highlight the need of factoring in p53 status in the design of combination treatments to potentiate the standard of care.

DNA damage activates the cGMP-AMP synthase (cGAS)–stimulator of interferon genes (STING) pathway, leading to type I interferon (IFN) production and upregulation of interferon-stimulated genes (ISGs) via transcriptional regulation by the interferon-stimulated gene factor 3 complex (ISGF3), comprising STAT1, STAT2, and IRF9 [82]. STING is epigenetically silenced in GBM neoplastic cells, although it is expressed in inflammatory and endothelial cells within the tumor microenvironment [83], while ISGs can also be induced in neoplastic cells by IFN-independent and STING-independent mechanisms [84]. In this study, we identified a highly correlated IFN response gene signature in GBM (TCGA data), which includes ISGF3 members such as IRF9 and STAT1, but not STING1. The dual role of type I IFNs in cancer biology is well established, with acute IFN treatment promoting anti-tumor immunity and neoplastic cell death—an approach with significant therapeutic potential for GBM [85]. This is supported by promising outcomes from recent clinical trials combining IFN-α [86] or IFN-β [87] with TMZ, particularly in tumors with unmethylated MGMT promoter. Conversely, chronic low-level IFN exposure can induce pro-tumor effects by fostering immunosuppression and resistance to RT and chemotherapy [82], and endogenous IFN/STAT1 activation has been associated with poor prognosis in proneural GBM [63]. In fact, a subset of ISGs associated with resistance to RT and DNA-damaging chemotherapy, termed IFN-related DNA damage resistance signature (IRDS), has been described in various cancers [57]. IRDS can be induced in cancer cells by prolonged exposure to low doses of type I IFN [88], among other stimuli. Consistently, we observed a robust upregulation of ISGs, including IRDS members, in response to TMZ and RT in GBM CSCs and SDCs. Future investigations are required to identify upstream regulators and evaluate the extent to which ISG activation in GBM cells contributes to therapeutic resistance to RT and TMZ. While selectively targeting IRDS components without impairing other critical immune functions remains challenging, our findings highlight the neoplastic cell intrinsic IFN response as a promising biomarker for predicting treatment outcomes. This insight paves the way for refining therapeutic strategies to address resistance mechanisms in GBM. Furthermore, type I IFN signaling has been inversely associated with cancer stemness across various cancers [89], aligning with our findings that differentiation of HF2303 CSCs is characterized by ISG upregulation. However, the absence of ISG induction in differentiated HF2927 cells underscores the heterogeneity in GBM CSC responses, emphasizing the need for further studies using larger panels of patient-derived models to assess the prevalence of endogenous ISG activation in differentiated CSCs and the effects of IFN treatment on CSC differentiation.

## Conclusions

Our integrated analysis of treatment response and differentiation in bulk patient-derived GBM cell populations provides valuable insights into the dynamic interplay between CSC and SDC sub-compartments. These findings validate prior research while offering novel perspectives for further in vivo investigation. Importantly, our results underscore the need for caution when extrapolating differentiation dynamics, treatment sensitivity, and molecular responses of CSCs across diverse GBM models. Notably, we identify the therapeutic potential of combining NF-κB inhibition with TMZ treatment and emphasize the critical importance of characterizing the prevalence and functional role of the neoplastic cell-intrinsic IFN response. Understanding its influence on CSC differentiation and resistance to DNA-damaging therapies is crucial in the context of the genomic and molecular heterogeneity of glioblastomas. These insights will help refine preclinical strategies and guide the development of more effective therapeutic approaches for this challenging malignancy.

## Methods

### Cell culture

Glioblastoma patient-derived cancer stem cell cultures were obtained from the live biobank collection at the Hermelin Brain Tumor Center, Henry Ford Hospital (Detroit, MI). These cells were derived from de-identified surgically resected glioblastoma specimens, collected with written informed consent, under a protocol approved by the HFH Institutional Review Board as previously described [90], in compliance with all relevant ethical regulations for research using human specimens. Cells were cultured in serum-free neurosphere media consisting of Dulbecco’s Modified Eagle Medium (DMEM)/F12 media (Invitrogen), N2 supplement (Gibco) and 0.5 mg/ml BSA, supplemented with growth factors 20 ng/ml EGF and 20 ng/ml bFGF (Peprotech) (NMGF), to select for CSCs [90]. CSC cultures were passaged in vitro and used prior to achieving passage 20. Identity of CSC lines was confirmed by comparing genotype with the patient germline using Short Tandem Repeat (STR) analysis. CSCs were dissociated and cultured for 2 weeks in three different conditions: NM lacking growth factors, and NMGF supplemented with 2% or 10% FBS (HyClone). Neural progenitor cells (NPCs) were derived from H9 human ESC, obtained from WiCell (Madison WI) as described [91] and differentiated in NMGF supplemented in 2% FBS [92].

### Reverse Phase Protein Arrays analysis

Cell lysates were obtained in triplicate for each cell line and growth conditions described (Fig 1B). The levels of 66 proteins/post-translational modifications in the cell lysates were analyzed by Reverse Phase Protein Arrays (RPPA) at the Mason’s Center for Applied Proteomics and Molecular Medicine (George Mason University), essentially as described previously [31,32]. Statistical analyses of RPPA normalized values were conducted in the R statistical environment (4.2.1). Heatmaps were generated by ComplexHeatmap package in R (2.12.1) [93]. Z-scores for each protein and post-translational modification were calculated based on population mean and standard deviation ((x-μ)/σ). Fold-change was calculated using the gTools package (3.9.3) and computed as follows: num/denom if num>denom, and as -denom/num otherwise. Dendrograms were produced using Spearman correlation as the distance method.

### Cell proliferation, radiation and temozolomide treatment

#### Cell proliferation

Cell proliferation curves were established by plating 1,000 cells/well into 96-well plates in NMGF (CSCs) or NMGF supplemented with 2% FBS (SDCs) and measuring cell viability after 1, 4, 6, 8, and 11 days in culture (n = 5). Wells were supplemented with fresh media every 3–4 days. Cell viability was measured using CellTiter-Glo Luminescent Cell Viability Assay (Promega), relative light units (RLU) were measured in a BioTek Cytation 3 plate reader. Doubling time (DT) was calculated using the formula DT = (t1-t2) ln2 / ln(RLU2/RLU1).

#### Temozolomide dose-response curves

CSCs and SDCs were plated in 96-well assay plates (3,000 cells/well), in quintuplicates and treated with 10 to 400 mM temozolomide (Schering-Plough) or DMSO control for 4 days. CellTiterGlo (Promega) was used to measure cell viability and dose-response curves were generated by non-linear fitting, IC50 doses and area above the curve (AAC) were calculated in GraphPad Prism (v.9.0).

#### Ionizing radiation treatment (RT)

CSC and SDC cells were dissociated, irradiated with 0, 2 or 4 Gy using a 5000 Ci Cesium (Cs-137) irradiator, and plated in triplicate at density of 3,000 cells/well in 96-well assay plates in the respective growth media and incubated under standard conditions for 5 days. Cell viability was assayed with CellTiter Glo (Promega). Surviving fractions at the two radiation doses were calculated and compared.

#### Tumorigenic potential of irradiated cells

Mouse experiments were performed under an approved Henry Ford Health Institutional Animal Care and Use Committee protocol (IACUC #1449) by properly trained personnel. Single cell suspensions of glioblastoma CSCs constitutively expressing firefly luciferase were irradiated with 0 (mock control) or 4Gy, as described above. Immediately following radiation or control treatment, 2x10^5^ cells/mouse were implanted intracranially in female NCRNU-M athymic nude mice (Taconic Farms), under anesthesia via ketamine/xylazine intraperitoneal injection, as described in detail [94]. To compare survival curves between mice implanted with CSCs treated with 0 Gy versus 4 Gy RT for each model, the required sample size was calculated using PASS Sample Size Software. The calculation was based on a one-tailed log-rank test with an alpha level of 0.10. With a sample size of 6 per group, there is 81% power to detect a difference in survival probabilities of 95% versus 5%. To account for possible losses, a total of 42 mice were used, n = 7/group except for HF2303 for which n = 6 mice were used for control and n = 8 for irradiated CSCs. Following surgery to implant CSCs, mice were kept warm using a heating pad until they regained consciousness, returned to the cages and for 48h observed every 3h for any signs of distress, in which case buprenorphine can be administered. Subsequently, mice continued to be monitored daily and were weighted 3 times a week by a technician blinded to the experimental groups. Tumor growth was monitored by noninvasive bioluminescence imaging using IVIS Spectrum In Vivo Imaging System (Caliper Life Sciences) once a week. The primary endpoint of this study is symptom-free survival, and no mice were excluded from the study. Within 6 hours after the observation of any symptoms associated with tumor burden, which happened within 275 days post-implant, all 42 mice were euthanized by >5% isoflurane overdose, and after no signs of palpebral reflexes and breathing were observed, mice were decapitated as a second assurance of death. The symptoms included weight loss greater than 20% of body mass, cranial protrusion, sickness, weakening, inactivity, anorexia, hunched posture, sunken eyes, ruffled coat, abnormal head tilt, seizures, or ataxia.

#### Sublethal temozolomide and radiation treatment for total RNA and genomic DNA isolation

*Temozolomide*. GBM CSCs or SDCs growing in their respective media were treated in triplicate with the respective TMZ IC40 concentrations or control DMSO for 4 days. *Radiation*: Cells were treated in triplicate with ionizing radiation (4Gy) or mock radiation control (0Gy) and cultured for 5 days. For all treatment groups, cells were harvested, total RNA isolated using RNeasy Mini Kit (Qiagen) and used for RNA sequencing. Genomic DNA was isolated using QIAamp DNA kit (Qiagen) and used for DNA methylation profiling.

### RNA sequencing

RNA quality assessment was performed using the Agilent RNA ScreenTape on the Agilent 2200 TapeStation. Library for RNA sequencing was prepared using TruSeq Stranded Total RNA LT (Illumina). 100 bps paired-end libraries were sequenced on the Illumina HiSeq 2500 following cBot clustering. Prior to alignment the sequencing adaptors were trimmed using FASTQ Toolkit v1.0 on Illumina’s BaseSpace portal. FastQC was used to generate quality metrics for assessment of FASTQ files [95]. RNA seq reads were aligned using TopHat v2.1.0 using default alignment parameters and GRCh38 reference genome [96,97]. After alignment, the mapped reads were further filtered by mappability score (MAPQ ≥ 10) and sorted by genomic position of the reads using samtools-0.1.19 [98]. PCR duplicate reads were processed and removed using rmdup function (options -S) in samtools. The cleaned BAM files were further analyzed using Integrative Genome Viewer (http://software.broadinstitute.org/software/igv/), processed with R function “FeatureCount” to quantify reads on various RNA types from GencodeV28 annotation. Differential gene expression analysis was performed using the non-parametric algorithm “NOISeq” in R **[99]**, with q = 0.8 and fold change >2 or <0.5 thresholds on TMM normalized data for treated vs. control comparisons, and q = 0.8 on RPKM normalized data for long term transcriptome changes in untreated SDCs vs CSCs. Gene set enrichment tests for differentially expressed gene lists were performed using Metascape 3.5, and adjusted p-value (q) < 0.05 [100].

### DNA methylation analysis

Genomic DNA was analyzed using Infinium Human Methylation 450K BeadChip system (Illumina), as described [91]. Raw methylation data was pre-processed for dye-bias normalization, detection p-value was calculated by comparing the signal intensity difference between array probes and a set of negative control probes on the array; probes with p-value greater than 0.01 filtered out. Beta values were calculated from pre-processed raw data using methylumi Bioconductor package in R [101]. Probes mapping to known SNPs and sex chromosomes were filtered out of dataset as recommended by Zhou et al. [102]. Beta values were corrected for batch effect using ComBat function within sva R package [103,104]. Probes were aligned to hg38 and annotated using Illumina methylation 450k manifest [105]. Differentially methylated positions between experimental groups (n = 3/group) were determined using champ.DMP function, and to determine differentially methylated regions we used the champ.DMR with Bumphunter method from the ChAMP package in ChAMPs R package [106] with raw p-values < 0.05, and adjusted p-value < 0.1. DMPs were annotated to genes using illuminaHg19 annotation employed by the ChAMPs package in R. Analyses to determine the intersect of DMPs, the genes mapped to DMPs and DEGs, within and between models, were carried out using GeneOverlap (version 1.34.0).

### Cellular oxygen consumption rate

Cellular oxygen consumption rate (OCR) was measured using a Seahorse XF Cell Mito Stress Test in a XFe24 Bioanalyzer (Agilent). Cells were plated at a concentration of 30,000 (HF2303) or 40,000 (HF2927) per well on poly-ornithine hydrobromide (Sigma # 4957) coated plates in NMGF media (CSC) or 2%FBS media (SDC) and treated for 4 days with DMSO or TMZ IC40 concentrations, prior to basal oxygen consumption measurements, performed according to the manufacturer’s instructions.

### Statistical analysis

Statistical analyses were performed using GraphPad Prism (v. 9.00) or R and are described in the results section for each experiment. Tumor growth in mouse PDX was evaluated by Kaplan-Meier survival curves, compared by the log-rank test with p<0.05 considered significant.

## Supporting information

S1 Fig Alteration in glioblastoma cancer stem cell signaling in response to growth factors withdrawal and differentiation in 2% FBS. A) Box plot representing changes in the levels of 66 proteins/PTM in response to 14-days culture in the absence of growth factors (NM), or upon serum differentiation (2%FBS), relative to CSC culture conditions (NMGF), using mean values from triplicate RPPA measurements for each of the 8 models. Plot represents n = 507 data points, after proteins/PTMs which were not detected under all three culture conditions were filtered out for each model.
(TIF)

S2 Fig MGMT promoter methylation. Heatmap representing β-values of 26 CpG sites mapping to MGMT promoter, for HF2303 and HF2927 CSC and SDCs in control, TMZ and RT treatment groups, in triplicate. Human astrocyte cell line was included as reference. The two probes widely employed in the clinic to determine MGMT promoter methylation status (“predictive probes”) indicate MGMT promoter hypermethylation in HF2927 and unmethylated status in HF2303 and human astrocytes cells. Differentiation status and treatment did not alter promoter methylation levels for either GBM line.
(TIF)

S3 Fig Alterations in cellular respiration in response to treatment. A) Basal (B) and maximum (M) relative oxygen consumption rate (OCR) for CSCs and SDCs measured after a 4-day treatment with TMZ, or 4 days after treatment with one 4 Gy radiation dose. OCR values were normalized to CSC (left panel) and SDC (right panel) HF2303 basal levels (%). Measurements for n = 2–3 repeats with means are shown. B) Reserve respiratory capacity was calculated as ratio of (mean maximum)/(mean basal) O2 consumption.
(TIF)

S4 Fig Short term treatment with TMZ and RT did not significantly alter DNA methylation pattern of glioblastoma CSCs and SDCs. Principal component analysis for b-values for all treatment groups in triplicates for each cell line and differentiation status: HF2303 CSC (A), HF2303 SDCs (B), HF2927 CSCs (C), and HF2927 SDCs (D).
(TIF)

S1 Table Reverse Phase Protein Array (RPPA) targets, values, signaling pathways. (XLSX)

S2 Table Differentially expressed gene lists. (XLSX)

S3 Table Interferon signaling correlation group in glioblastoma (TCGA). (XLSX)

S4 Table Differentially methylated probes. (XLSX)

## References

[1] Louis DN, Perry A, Wesseling P, Brat DJ, Cree IA, Figarella-Branger D, et al. The 2021 WHO Classification of Tumors of the Central Nervous System: a summary. Neuro-oncology. 2021;23(8):1231–51. Epub 2021/06/30. doi: 10.1093/neuonc/noab106 ; PubMed Central PMCID: PMC8328013. 34185076 PMC8328013

[2] Marko NF, Weil RJ, Schroeder JL, Lang FF, Suki D, Sawaya RE. Extent of resection of glioblastoma revisited: personalized survival modeling facilitates more accurate survival prediction and supports a maximum-safe-resection approach to surgery. Journal of clinical oncology: official journal of the American Society of Clinical Oncology. 2014;32(8):774–82. Epub 20140210. doi: 10.1200/JCO.2013.51.8886 ; PubMed Central PMCID: PMC4876349. 24516010 PMC4876349

[3] Stupp R, Mason WP, van den Bent MJ, Weller M, Fisher B, Taphoorn MJ, et al. Radiotherapy plus concomitant and adjuvant temozolomide for glioblastoma. The New England journal of medicine. 2005;352(10):987–96. doi: 10.1056/NEJMoa043330 . 15758009

[4] Marenco-Hillembrand L, Wijesekera O, Suarez-Meade P, Mampre D, Jackson C, Peterson J, et al. Trends in glioblastoma: outcomes over time and type of intervention: a systematic evidence based analysis. Journal of neuro-oncology. 2020;147(2):297–307. Epub 2020/03/12. doi: 10.1007/s11060-020-03451-6 . 32157552

[5] Hegi ME, Diserens A-C, Gorlia T, Hamou M-F, de Tribolet N, Weller M, et al. MGMT Gene Silencing and Benefit from Temozolomide in Glioblastoma. The New England journal of medicine. 2005;352(10):997–1003. doi: 10.1056/NEJMoa043331 15758010

[6] Chen X, Zhang M, Gan H, Wang H, Lee JH, Fang D, et al. A novel enhancer regulates MGMT expression and promotes temozolomide resistance in glioblastoma. Nat Commun. 2018;9(1):2949. Epub 20180727. doi: 10.1038/s41467-018-05373-4 ; PubMed Central PMCID: PMC6063898. 30054476 PMC6063898

[7] Lavon I, Fuchs D, Zrihan D, Efroni G, Zelikovitch B, Fellig Y, et al. Novel mechanism whereby nuclear factor kappaB mediates DNA damage repair through regulation of O(6)-methylguanine-DNA-methyltransferase. Cancer Res. 2007;67(18):8952–9. doi: 10.1158/0008-5472.CAN-06-3820 . 17875738

[8] Sanchez-Vega F, Mina M, Armenia J, Chatila WK, Luna A, La KC, et al. Oncogenic Signaling Pathways in The Cancer Genome Atlas. Cell. 2018;173(2):321–37. e10. doi: 10.1016/j.cell.2018.03.035 29625050 PMC6070353

[9] Olson JJ, Nayak L, Ormond DR, Wen PY, Kalkanis SN, Ryken TC. The role of targeted therapies in the management of progressive glioblastoma: a systematic review and evidence-based clinical practice guideline. Journal of neuro-oncology. 2014;118:557–99. doi: 10.1007/s11060-013-1339-4 24740195

[10] Johnson BE, Mazor T, Hong C, Barnes M, Aihara K, McLean CY, et al. Mutational analysis reveals the origin and therapy-driven evolution of recurrent glioma. Science. 2014;343(6167):189–93. Epub 2013/12/18. doi: 10.1126/science.1239947 ; PubMed Central PMCID: PMC3998672. 24336570 PMC3998672

[11] deCarvalho AC, Kim H, Poisson LM, Winn ME, Mueller C, Cherba D, et al. Discordant inheritance of chromosomal and extrachromosomal DNA elements contributes to dynamic disease evolution in glioblastoma. Nat Genet. 2018;50(5):708–17. Epub April 23. doi: 10.1038/s41588-018-0105-0 ; PubMed Central PMCID: PMC5934307. 29686388 PMC5934307

[12] Neftel C, Laffy J, Filbin MG, Hara T, Shore ME, Rahme GJ, et al. An Integrative Model of Cellular States, Plasticity, and Genetics for Glioblastoma. Cell. 2019;178(4):835–49 e21. Epub 20190718. doi: 10.1016/j.cell.2019.06.024 ; PubMed Central PMCID: PMC6703186. 31327527 PMC6703186

[13] Yabo YA, Niclou SP, Golebiewska A. Cancer cell heterogeneity and plasticity: A paradigm shift in glioblastoma. Neuro-oncology. 2022;24(5):669–82. doi: 10.1093/neuonc/noab269 ; PubMed Central PMCID: PMC9071273. 34932099 PMC9071273

[14] Reinartz R, Wang S, Kebir S, Silver DJ, Wieland A, Zheng T, et al. Functional Subclone Profiling for Prediction of Treatment-Induced Intratumor Population Shifts and Discovery of Rational Drug Combinations in Human Glioblastoma. Clin Cancer Res. 2017;23(2):562–74. Epub 20160812. doi: 10.1158/1078-0432.CCR-15-2089 ; PubMed Central PMCID: PMC5241221. 27521447 PMC5241221

[15] Suvà ML, Rheinbay E, Gillespie SM, Patel AP, Wakimoto H, Rabkin SD, et al. Reconstructing and reprogramming the tumor-propagating potential of glioblastoma stem-like cells. Cell. 2014;157(3):580–94. doi: 10.1016/j.cell.2014.02.030 24726434 PMC4004670

[16] Galli R, Binda E, Orfanelli U, Cipelletti B, Gritti A, De Vitis S, et al. Isolation and characterization of tumorigenic, stem-like neural precursors from human glioblastoma. Cancer Res. 2004;64(19):7011–21. doi: 10.1158/0008-5472.CAN-04-1364 . 15466194

[17] Vescovi AL, Galli R, Reynolds BA. Brain tumour stem cells. Nat Rev Cancer. 2006;6(6):425–36. doi: 10.1038/nrc1889 . 16723989

[18] Ignatova TN, Kukekov VG, Laywell ED, Suslov ON, Vrionis FD, Steindler DA. Human cortical glial tumors contain neural stem-like cells expressing astroglial and neuronal markers in vitro. Glia. 2002;39(3):193–206. doi: 10.1002/glia.10094 . 12203386

[19] Hemmati HD, Nakano I, Lazareff JA, Masterman-Smith M, Geschwind DH, Bronner-Fraser M, et al. Cancerous stem cells can arise from pediatric brain tumors. Proc Natl Acad Sci U S A. 2003;100(25):15178–83. doi: 10.1073/pnas.2036535100 . 14645703 PMC299944

[20] Shen Y, Grisdale CJ, Islam SA, Bose P, Lever J, Zhao EY, et al. Comprehensive genomic profiling of glioblastoma tumors, BTICs, and xenografts reveals stability and adaptation to growth environments. Proc Natl Acad Sci U S A. 2019;116(38):19098–108. Epub 2019/09/01. doi: 10.1073/pnas.1813495116 ; PubMed Central PMCID: PMC6754609. 31471491 PMC6754609

[21] Woo XY, Giordano J, Srivastava A, Zhao ZM, Lloyd MW, de Bruijn R, et al. Conservation of copy number profiles during engraftment and passaging of patient-derived cancer xenografts. Nat Genet. 2021;53(1):86–99. Epub 20210107. doi: 10.1038/s41588-020-00750-6 ; PubMed Central PMCID: PMC7808565. 33414553 PMC7808565

[22] Quartararo CE, Reznik E, deCarvalho AC, Mikkelsen T, Stockwell BR. High-Throughput Screening of Patient-Derived Cultures Reveals Potential for Precision Medicine in Glioblastoma. ACS medicinal chemistry letters. 2015;6(8):948–52. doi: 10.1021/acsmedchemlett.5b00128 ; PubMed Central PMCID: PMC4538440. 26288699 PMC4538440

[23] Brennan CW, Verhaak RG, McKenna A, Campos B, Noushmehr H, Salama SR, et al. The somatic genomic landscape of glioblastoma. Cell. 2013;155(2):462–77. Epub 2013/10/15. doi: 10.1016/j.cell.2013.09.034 ; PubMed Central PMCID: PMC3910500. 24120142 PMC3910500

[24] Mendoza MC, Er EE, Blenis J. The Ras-ERK and PI3K-mTOR pathways: cross-talk and compensation. Trends Biochem Sci. 2011;36(6):320–8. Epub 20110430. doi: 10.1016/j.tibs.2011.03.006 ; PubMed Central PMCID: PMC3112285. 21531565 PMC3112285

[25] Ye LF, Reznik E, Korn JM, Lin F, Yang G, Malesky K, et al. Patient-derived glioblastoma cultures as a tool for small-molecule drug discovery. Oncotarget. 2020;11(4):443–51. Epub 2020/02/18. doi: 10.18632/oncotarget.27457 ; PubMed Central PMCID: PMC6996910. 32064048 PMC6996910

[26] Berezovsky AD, Poisson LM, Cherba D, Webb CP, Transou AD, Lemke NW, et al. Sox2 promotes malignancy in glioblastoma by regulating plasticity and astrocytic differentiation. Neoplasia. 2014;16(3):193–206 e25. Epub 2014/04/15. doi: 10.1016/j.neo.2014.03.006 . 24726753 PMC4094829

[27] Zheng X, Baker H, Hancock WS, Fawaz F, McCaman M, Pungor E Jr. Proteomic analysis for the assessment of different lots of fetal bovine serum as a raw material for cell culture. Part IV. Application of proteomics to the manufacture of biological drugs. Biotechnol Prog. 2006;22(5):1294–300. doi: 10.1021/bp060121o . 17022666

[28] Lee J, Kotliarova S, Kotliarov Y, Li A, Su Q, Donin NM, et al. Tumor stem cells derived from glioblastomas cultured in bFGF and EGF more closely mirror the phenotype and genotype of primary tumors than do serum-cultured cell lines. Cancer cell. 2006;9(5):391–403. doi: 10.1016/j.ccr.2006.03.030 . 16697959

[29] Shaltouki A, Peng J, Liu Q, Rao MS, Zeng X. Efficient generation of astrocytes from human pluripotent stem cells in defined conditions. Stem Cells. 2013;31(5):941–52. doi: 10.1002/stem.1334 . 23341249

[30] Tcw J, Wang M, Pimenova AA, Bowles KR, Hartley BJ, Lacin E, et al. An Efficient Platform for Astrocyte Differentiation from Human Induced Pluripotent Stem Cells. Stem cell reports. 2017;9(2):600–14. Epub 20170727. doi: 10.1016/j.stemcr.2017.06.018 ; PubMed Central PMCID: PMC5550034. 28757165 PMC5550034

[31] Mueller C, deCarvalho AC, Mikkelsen T, Lehman NL, Calvert V, Espina V, et al. Glioblastoma cell enrichment is critical for analysis of phosphorylated drug targets and proteomic-genomic correlations. Cancer Res. 2014;74(3):818–28. Epub 2013/12/19. doi: 10.1158/0008-5472.CAN-13-2172 . 24346432

[32] Pin E, Federici G, Petricoin EF, 3rd. Preparation and use of reverse protein microarrays. Current protocols in protein science / editorial board, John E Coligan [et al]. 2014;75:Unit 27 7. doi: 10.1002/0471140864.ps2707s75 . 24510676

[33] Kodaira K, Imada M, Goto M, Tomoyasu A, Fukuda T, Kamijo R, et al. Purification and identification of a BMP-like factor from bovine serum. Biochem Biophys Res Commun. 2006;345(3):1224–31. Epub 20060515. doi: 10.1016/j.bbrc.2006.05.045 . 16716261

[34] Yang G, Murashige DS, Humphrey SJ, James DE. A Positive Feedback Loop between Akt and mTORC2 via SIN1 Phosphorylation. Cell reports. 2015;12(6):937–43. Epub 20150730. doi: 10.1016/j.celrep.2015.07.016 . 26235620

[35] Efeyan A, Sabatini DM. mTOR and cancer: many loops in one pathway. Curr Opin Cell Biol. 2010;22(2):169–76. Epub 20091127. doi: 10.1016/j.ceb.2009.10.007 ; PubMed Central PMCID: PMC2854285. 19945836 PMC2854285

[36] Copp J, Manning G, Hunter T. TORC-specific phosphorylation of mammalian target of rapamycin (mTOR): phospho-Ser2481 is a marker for intact mTOR signaling complex 2. Cancer Res. 2009;69(5):1821–7. Epub 20090224. doi: 10.1158/0008-5472.CAN-08-3014 ; PubMed Central PMCID: PMC2652681. 19244117 PMC2652681

[37] Boutouja F, Stiehm CM, Platta HW. mTOR: A Cellular Regulator Interface in Health and Disease. Cells. 2019;8(1). Epub 20190102. doi: 10.3390/cells8010018 ; PubMed Central PMCID: PMC6356367. 30609721 PMC6356367

[38] Ovens AJ, Scott JW, Langendorf CG, Kemp BE, Oakhill JS, Smiles WJ. Post-Translational Modifications of the Energy Guardian AMP-Activated Protein Kinase. Int J Mol Sci. 2021;22(3). Epub 20210127. doi: 10.3390/ijms22031229 ; PubMed Central PMCID: PMC7866021. 33513781 PMC7866021

[39] Dite TA, Ling NXY, Scott JW, Hoque A, Galic S, Parker BL, et al. The autophagy initiator ULK1 sensitizes AMPK to allosteric drugs. Nat Commun. 2017;8(1):571. Epub 20170918. doi: 10.1038/s41467-017-00628-y ; PubMed Central PMCID: PMC5603566. 28924239 PMC5603566

[40] Cirotti C, Contadini C, Barila D. SRC Kinase in Glioblastoma News from an Old Acquaintance. Cancers. 2020;12(6). Epub 20200612. doi: 10.3390/cancers12061558 ; PubMed Central PMCID: PMC7352599. 32545574 PMC7352599

[41] Okada M. Regulation of the SRC family kinases by Csk. Int J Biol Sci. 2012;8(10):1385–97. Epub 20121101. doi: 10.7150/ijbs.5141 ; PubMed Central PMCID: PMC3492796. 23139636 PMC3492796

[42] Irtegun S, Wood RJ, Ormsby AR, Mulhern TD, Hatters DM. Tyrosine 416 is phosphorylated in the closed, repressed conformation of c-Src. PloS one. 2013;8(7):e71035. Epub 20130726. doi: 10.1371/journal.pone.0071035 ; PubMed Central PMCID: PMC3724807. 23923048 PMC3724807

[43] Metz HE, Houghton AM. Insulin receptor substrate regulation of phosphoinositide 3-kinase. Clin Cancer Res. 2011;17(2):206–11. Epub 20101021. doi: 10.1158/1078-0432.CCR-10-0434 . 20966354

[44] Li J, Choi E, Yu H, Bai XC. Structural basis of the activation of type 1 insulin-like growth factor receptor. Nat Commun. 2019;10(1):4567. Epub 20191008. doi: 10.1038/s41467-019-12564-0 ; PubMed Central PMCID: PMC6783537. 31594955 PMC6783537

[45] Shah OJ, Hunter T. Turnover of the active fraction of IRS1 involves raptor-mTOR- and S6K1-dependent serine phosphorylation in cell culture models of tuberous sclerosis. Mol Cell Biol. 2006;26(17):6425–34. doi: 10.1128/MCB.01254-05 ; PubMed Central PMCID: PMC1592824. 16914728 PMC1592824

[46] Rena G, Prescott AR, Guo S, Cohen P, Unterman TG. Roles of the forkhead in rhabdomyosarcoma (FKHR) phosphorylation sites in regulating 14-3-3 binding, transactivation and nuclear targetting. Biochem J. 2001;354(Pt 3):605–12. doi: 10.1042/0264-6021:3540605 ; PubMed Central PMCID: PMC1221692. 11237865 PMC1221692

[47] Li A, Walling J, Kotliarov Y, Center A, Steed ME, Ahn SJ, et al. Genomic changes and gene expression profiles reveal that established glioma cell lines are poorly representative of primary human gliomas. Mol Cancer Res. 2008;6(1):21–30. doi: 10.1158/1541-7786.MCR-07-0280 . 18184972

[48] Mighell TL, Evans-Dutson S, O’Roak BJ. A Saturation Mutagenesis Approach to Understanding PTEN Lipid Phosphatase Activity and Genotype-Phenotype Relationships. Am J Hum Genet. 2018;102(5):943–55. Epub 20180426. doi: 10.1016/j.ajhg.2018.03.018 ; PubMed Central PMCID: PMC5986715. 29706350 PMC5986715

[49] Vazquez F, Ramaswamy S, Nakamura N, Sellers WR. Phosphorylation of the PTEN tail regulates protein stability and function. Mol Cell Biol. 2000;20(14):5010–8. doi: 10.1128/MCB.20.14.5010-5018.2000 ; PubMed Central PMCID: PMC85951. 10866658 PMC85951

[50] Rubio-Camacho M, Encinar JA, Martinez-Tome MJ, Esquembre R, Mateo CR. The Interaction of Temozolomide with Blood Components Suggests the Potential Use of Human Serum Albumin as a Biomimetic Carrier for the Drug. Biomolecules. 2020;10(7). Epub 20200709. doi: 10.3390/biom10071015 ; PubMed Central PMCID: PMC7408562. 32659914 PMC7408562

[51] Manem VS, Lambie M, Smith I, Smirnov P, Kofia V, Freeman M, et al. Modeling Cellular Response in Large-Scale Radiogenomic Databases to Advance Precision Radiotherapy. Cancer Res. 2019;79(24):6227–37. Epub 2019/09/29. doi: 10.1158/0008-5472.CAN-19-0179 ; PubMed Central PMCID: PMC8128135. 31558563 PMC8128135

[52] Sancho P, Barneda D, Heeschen C. Hallmarks of cancer stem cell metabolism. Br J Cancer. 2016;114(12):1305–12. Epub 20160524. doi: 10.1038/bjc.2016.152 ; PubMed Central PMCID: PMC4984474. 27219018 PMC4984474

[53] Yamatani Y, Nakai K. Comprehensive comparison of gene expression diversity among a variety of human stem cells. NAR Genom Bioinform. 2022;4(4):lqac087. Epub 20221129. doi: 10.1093/nargab/lqac087 ; PubMed Central PMCID: PMC9706419. 36458020 PMC9706419

[54] Bodzioch M, Lapicka-Bodzioch K, Zapala B, Kamysz W, Kiec-Wilk B, Dembinska-Kiec A. Evidence for potential functionality of nuclearly-encoded humanin isoforms. Genomics. 2009;94(4):247–56. Epub 20090527. doi: 10.1016/j.ygeno.2009.05.006 . 19477263

[55] Fischer M. Census and evaluation of p53 target genes. Oncogene. 2017;36(28):3943–56. Epub 20170313. doi: 10.1038/onc.2016.502 ; PubMed Central PMCID: PMC5511239. 28288132 PMC5511239

[56] Gimple RC, Kidwell RL, Kim LJY, Sun T, Gromovsky AD, Wu Q, et al. Glioma Stem Cell-Specific Superenhancer Promotes Polyunsaturated Fatty-Acid Synthesis to Support EGFR Signaling. Cancer discovery. 2019;9(9):1248–67. Epub 20190614. doi: 10.1158/2159-8290.CD-19-0061 ; PubMed Central PMCID: PMC6785242. 31201181 PMC6785242

[57] Weichselbaum RR, Ishwaran H, Yoon T, Nuyten DS, Baker SW, Khodarev N, et al. An interferon-related gene signature for DNA damage resistance is a predictive marker for chemotherapy and radiation for breast cancer. Proc Natl Acad Sci U S A. 2008;105(47):18490–5. Epub 20081110. doi: 10.1073/pnas.0809242105 ; PubMed Central PMCID: PMC2587578. 19001271 PMC2587578

[58] Tanner SM, Austin JL, Leone G, Rush LJ, Plass C, Heinonen K, et al. BAALC, the human member of a novel mammalian neuroectoderm gene lineage, is implicated in hematopoiesis and acute leukemia. Proc Natl Acad Sci U S A. 2001;98(24):13901–6. Epub 2001/11/15. doi: 10.1073/pnas.241525498 ; PubMed Central PMCID: PMC61139. 11707601 PMC61139

[59] Ferrandez E, Gutierrez O, Segundo DS, Fernandez-Luna JL. NFkappaB activation in differentiating glioblastoma stem-like cells is promoted by hyaluronic acid signaling through TLR4. Sci Rep. 2018;8(1):6341. Epub 20180420. doi: 10.1038/s41598-018-24444-6 ; PubMed Central PMCID: PMC5910430. 29679017 PMC5910430

[60] Bochukova EG, Lawler K, Croizier S, Keogh JM, Patel N, Strohbehn G, et al. A Transcriptomic Signature of the Hypothalamic Response to Fasting and BDNF Deficiency in Prader-Willi Syndrome. Cell reports. 2018;22(13):3401–8. doi: 10.1016/j.celrep.2018.03.018 ; PubMed Central PMCID: PMC5896230. 29590610 PMC5896230

[61] Amsden H, Kourko O, Roth M, Gee K. Antiviral Activities of Interleukin-27: A Partner for Interferons? Front Immunol. 2022;13:902853. Epub 20220510. doi: 10.3389/fimmu.2022.902853 ; PubMed Central PMCID: PMC9134790. 35634328 PMC9134790

[62] Cerami E, Gao J, Dogrusoz U, Gross BE, Sumer SO, Aksoy BA, et al. The cBio cancer genomics portal: an open platform for exploring multidimensional cancer genomics data. Cancer discovery. 2012;2(5):401–4. Epub 2012/05/17. doi: 10.1158/2159-8290.CD-12-0095 . 22588877 PMC3956037

[63] Duarte CW, Willey CD, Zhi D, Cui X, Harris JJ, Vaughan LK, et al. Expression signature of IFN/STAT1 signaling genes predicts poor survival outcome in glioblastoma multiforme in a subtype-specific manner. PloS one. 2012;7(1):e29653. Epub 20120105. doi: 10.1371/journal.pone.0029653 ; PubMed Central PMCID: PMC3252343. 22242177 PMC3252343

[64] Engeland K. Cell cycle arrest through indirect transcriptional repression by p53: I have a DREAM. Cell Death Differ. 2018;25(1):114–32. Epub 20171110. doi: 10.1038/cdd.2017.172 ; PubMed Central PMCID: PMC5729532. 29125603 PMC5729532

[65] Baskar R, Dai J, Wenlong N, Yeo R, Yeoh KW. Biological response of cancer cells to radiation treatment. Front Mol Biosci. 2014;1:24. Epub 20141117. doi: 10.3389/fmolb.2014.00024 ; PubMed Central PMCID: PMC4429645. 25988165 PMC4429645

[66] Fischer M, Grossmann P, Padi M, DeCaprio JA. Integration of TP53, DREAM, MMB-FOXM1 and RB-E2F target gene analyses identifies cell cycle gene regulatory networks. Nucleic acids research. 2016;44(13):6070–86. Epub 20160608. doi: 10.1093/nar/gkw523 ; PubMed Central PMCID: PMC4994865. 27280975 PMC4994865

[67] Fischer M, Schade AE, Branigan TB, Muller GA, DeCaprio JA. Coordinating gene expression during the cell cycle. Trends Biochem Sci. 2022;47(12):1009–22. Epub 20220711. doi: 10.1016/j.tibs.2022.06.007 . 35835684

[68] Marceau AH, Brison CM, Nerli S, Arsenault HE, McShan AC, Chen E, et al. An order-to-disorder structural switch activates the FoxM1 transcription factor. Elife. 2019;8. Epub 20190528. doi: 10.7554/eLife.46131 ; PubMed Central PMCID: PMC6538375. 31134895 PMC6538375

[69] Rajan P, McKay RD. Multiple routes to astrocytic differentiation in the CNS. J Neurosci. 1998;18(10):3620–9. doi: 10.1523/JNEUROSCI.18-10-03620.1998 ; PubMed Central PMCID: PMC6793143. 9570793 PMC6793143

[70] Srikanth M, Kim J, Das S, Kessler JA. BMP signaling induces astrocytic differentiation of clinically derived oligodendroglioma propagating cells. Mol Cancer Res. 2014;12(2):283–94. Epub 20131122. doi: 10.1158/1541-7786.MCR-13-0349 ; PubMed Central PMCID: PMC4006982. 24269952 PMC4006982

[71] Zhang Y, Kwok-Shing Ng P, Kucherlapati M, Chen F, Liu Y, Tsang YH, et al. A Pan-Cancer Proteogenomic Atlas of PI3K/AKT/mTOR Pathway Alterations. Cancer cell. 2017;31(6):820–32 e3. Epub 20170518. doi: 10.1016/j.ccell.2017.04.013 ; PubMed Central PMCID: PMC5502825. 28528867 PMC5502825

[72] Bao S, Wu Q, McLendon RE, Hao Y, Shi Q, Hjelmeland AB, et al. Glioma stem cells promote radioresistance by preferential activation of the DNA damage response. Nature. 2006;444(7120):756–60. Epub 2006/10/20. doi: 10.1038/nature05236 . 17051156

[73] Aasland D, Götzinger L, Hauck L, Berte N, Meyer J, Effenberger M, et al. Temozolomide induces senescence and repression of DNA repair pathways in glioblastoma cells via activation of ATR–CHK1, p21, and NF-κB. Cancer research. 2019;79(1):99–113. 30361254 10.1158/0008-5472.CAN-18-1733

[74] Avci NG, Ebrahimzadeh-Pustchi S, Akay YM, Esquenazi Y, Tandon N, Zhu J-J, et al. NF-κB inhibitor with Temozolomide results in significant apoptosis in glioblastoma via the NF-κB (p65) and actin cytoskeleton regulatory pathways. Scientific Reports. 2020;10(1):13352. 32770097 10.1038/s41598-020-70392-5 PMC7414229

[75] Roth P, Gorlia T, Reijneveld JC, de Vos F, Idbaih A, Frenel JS, et al. Marizomib for patients with newly diagnosed glioblastoma: A randomized phase 3 trial. Neuro-oncology. 2024;26(9):1670–82. doi: 10.1093/neuonc/noae053 ; PubMed Central PMCID: PMC11376448. 38502052 PMC11376448

[76] Galinski B, Luxemburg M, Landesman Y, Pawel B, Johnson KJ, Master SR, et al. XPO1 inhibition with selinexor synergizes with proteasome inhibition in neuroblastoma by targeting nuclear export of IkB. Translational oncology. 2021;14(8):101114. Epub 20210509. doi: 10.1016/j.tranon.2021.101114 ; PubMed Central PMCID: PMC8131731. 33975179 PMC8131731

[77] Villa GR, Hulce JJ, Zanca C, Bi J, Ikegami S, Cahill GL, et al. An LXR-Cholesterol Axis Creates a Metabolic Co-Dependency for Brain Cancers. Cancer cell. 2016;30(5):683–93. Epub 20161013. doi: 10.1016/j.ccell.2016.09.008 ; PubMed Central PMCID: PMC5479636. 27746144 PMC5479636

[78] Wiese S, Karus M, Faissner A. Astrocytes as a source for extracellular matrix molecules and cytokines. Front Pharmacol. 2012;3:120. Epub 20120626. doi: 10.3389/fphar.2012.00120 ; PubMed Central PMCID: PMC3382726. 22740833 PMC3382726

[79] Segerman A, Niklasson M, Haglund C, Bergstrom T, Jarvius M, Xie Y, et al. Clonal Variation in Drug and Radiation Response among Glioma-Initiating Cells Is Linked to Proneural-Mesenchymal Transition. Cell Rep. 2016;17(11):2994–3009. doi: 10.1016/j.celrep.2016.11.056 . 27974212

[80] Zielske SP. Epigenetic DNA methylation in radiation biology: on the field or on the sidelines? J Cell Biochem. 2015;116(2):212–7. doi: 10.1002/jcb.24959 . 25186310

[81] Hanel W, Marchenko N, Xu S, Xiaofeng Yu S, Weng W, Moll U. Two hot spot mutant p53 mouse models display differential gain of function in tumorigenesis. Cell Death & Differentiation. 2013;20(7):898–909. doi: 10.1038/cdd.2013.17 23538418 PMC3679454

[82] Cheon H, Wang Y, Wightman SM, Jackson MW, Stark GR. How cancer cells make and respond to interferon-I. Trends in cancer. 2023;9(1):83–92. doi: 10.1016/j.trecan.2022.09.003 36216730 PMC9797472

[83] Low JT, Chandramohan V, Bowie ML, Brown MC, Waitkus MS, Briley A, et al. Epigenetic STING silencing is developmentally conserved in gliomas and can be rescued by methyltransferase inhibition. Cancer cell. 2022;40(5):439–40. Epub 20220428. doi: 10.1016/j.ccell.2022.04.009 . 35487217

[84] Goedegebuure RSA, Kleibeuker EA, Buffa FM, Castricum KCM, Haider S, Schulkens IA, et al. Interferon- and STING-independent induction of type I interferon stimulated genes during fractionated irradiation. J Exp Clin Cancer Res. 2021;40(1):161. Epub 20210508. doi: 10.1186/s13046-021-01962-2 ; PubMed Central PMCID: PMC8106844. 33964942 PMC8106844

[85] Lim J, Kang I, La J, Ku KB, Kang BH, Kim Y, et al. Harnessing type I interferon-mediated immunity to target malignant brain tumors. Front Immunol. 2023;14:1203929. Epub 20230525. doi: 10.3389/fimmu.2023.1203929 ; PubMed Central PMCID: PMC10247981. 37304294 PMC10247981

[86] Guo C, Yang Q, Xu P, Deng M, Jiang T, Cai L, et al. Adjuvant temozolomide chemotherapy with or without interferon Alfa among patients with newly diagnosed high-grade gliomas: a randomized clinical trial. JAMA Network Open. 2023;6(1):e2253285–e. doi: 10.1001/jamanetworkopen.2022.53285 36705923 PMC11839150

[87] Motomura K, Natsume A, Kishida Y, Higashi H, Kondo Y, Nakasu Y, et al. Benefits of interferon‐β and temozolomide combination therapy for newly diagnosed primary glioblastoma with the unmethylated MGMT promoter: A multicenter study. Cancer. 2011;117(8):1721–30. 21472719 10.1002/cncr.25637

[88] Khodarev NN, Beckett M, Labay E, Darga T, Roizman B, Weichselbaum RR. STAT1 is overexpressed in tumors selected for radioresistance and confers protection from radiation in transduced sensitive cells. Proceedings of the National Academy of Sciences. 2004;101(6):1714–9. doi: 10.1073/pnas.0308102100 14755057 PMC341831

[89] Miranda A, Hamilton PT, Zhang AW, Pattnaik S, Becht E, Mezheyeuski A, et al. Cancer stemness, intratumoral heterogeneity, and immune response across cancers. Proc Natl Acad Sci U S A. 2019;116(18):9020–9. Epub 20190417. doi: 10.1073/pnas.1818210116 ; PubMed Central PMCID: PMC6500180. 30996127 PMC6500180

[90] Hasselbach LA, Irtenkauf SM, Lemke NW, Nelson KK, Berezovsky AD, Carlton ET, et al. Optimization of High Grade Glioma Cell Culture from Surgical Specimens for Use in Clinically Relevant Animal Models and 3D Immunochemistry. J Vis Exp. 2014;83:e51088. Epub 2014/01/17. doi: 10.3791/51088 . 24429465 PMC4089397

[91] Senut MC, Sen A, Cingolani P, Shaik A, Land SJ, Ruden DM. Lead exposure disrupts global DNA methylation in human embryonic stem cells and alters their neuronal differentiation. Toxicol Sci. 2014;139(1):142–61. Epub 2014/02/13. doi: 10.1093/toxsci/kfu028 ; PubMed Central PMCID: PMC4023291. 24519525 PMC4023291

[92] Haidet-Phillips AM, Hester ME, Miranda CJ, Meyer K, Braun L, Frakes A, et al. Astrocytes from familial and sporadic ALS patients are toxic to motor neurons. Nat Biotechnol. 2011;29(9):824–8. Epub 2011/08/13. doi: 10.1038/nbt.1957 ; PubMed Central PMCID: PMC3170425. 21832997 PMC3170425

[93] Gu Z, Eils R, Schlesner M. Complex heatmaps reveal patterns and correlations in multidimensional genomic data. Bioinformatics. 2016;32(18):2847–9. Epub 20160520. doi: 10.1093/bioinformatics/btw313 . 27207943

[94] Irtenkauf SM, Sobiechowski S, Hasselbach LA, Nelson KK, Transou AD, Carlton ET, et al. Optimization of Glioblastoma Mouse Orthotopic Xenograft Models for Translational Research. Comparative medicine. 2017;67(4):300–14. Epub May 16. doi: no_doi/1494959945479 ; PubMed Central PMCID: PMC5557202. 28830577 PMC5557202

[95] Andrews S. FastQC A Quality Control Tool for High Throughput Sequence Data. Available online at: http://www.bioinformatics.babraham.ac.uk/projects/fastqc2010.

[96] Kim D, Pertea G, Trapnell C, Pimentel H, Kelley R, Salzberg SL. TopHat2: accurate alignment of transcriptomes in the presence of insertions, deletions and gene fusions. Genome Biol. 2013;14(4):R36. Epub 2013/04/27. doi: 10.1186/gb-2013-14-4-r36 ; PubMed Central PMCID: PMC4053844. 23618408 PMC4053844

[97] Trapnell C, Pachter L, Salzberg SL. TopHat: discovering splice junctions with RNA-Seq. Bioinformatics. 2009;25(9):1105–11. Epub 2009/03/18. doi: 10.1093/bioinformatics/btp120 ; PubMed Central PMCID: PMC2672628. 19289445 PMC2672628

[98] Li H, Handsaker B, Wysoker A, Fennell T, Ruan J, Homer N, et al. The Sequence Alignment/Map format and SAMtools. Bioinformatics. 2009;25(16):2078–9. Epub 2009/06/10. doi: 10.1093/bioinformatics/btp352 ; PubMed Central PMCID: PMC2723002. 19505943 PMC2723002

[99] Tarazona S, Furio-Tari P, Turra D, Pietro AD, Nueda MJ, Ferrer A, et al. Data quality aware analysis of differential expression in RNA-seq with NOISeq R/Bioc package. Nucleic acids research. 2015;43(21):e140. Epub 2015/07/18. doi: 10.1093/nar/gkv711 ; PubMed Central PMCID: PMC4666377. 26184878 PMC4666377

[100] Zhou Y, Zhou B, Pache L, Chang M, Khodabakhshi AH, Tanaseichuk O, et al. Metascape provides a biologist-oriented resource for the analysis of systems-level datasets. Nat Commun. 2019;10(1):1523. Epub 2019/04/05. doi: 10.1038/s41467-019-09234-6 ; PubMed Central PMCID: PMC6447622. 30944313 PMC6447622

[101] Davis S, Du P, Bilke S, Triche T Jr, Bootwalla M. methylumi: Handle Illumina methylation data. R package version 2.30.0. doi: 10.18129/B9.bioc.methylumi 2019.

[102] Zhou W, Laird PW, Shen H. Comprehensive characterization, annotation and innovative use of Infinium DNA methylation BeadChip probes. Nucleic acids research. 2017;45(4):e22. Epub 2016/12/08. doi: 10.1093/nar/gkw967 ; PubMed Central PMCID: PMC5389466. 27924034 PMC5389466

[103] Leek JT, Johnson WE, Parker HS, Fertig EJ, Jaffe AE, Storey JD, et al. sva: Surrogate Variable Analysis. R package version 3.32.1. 2019.

[104] Johnson WE, Li C, Rabinovic A. Adjusting batch effects in microarray expression data using empirical Bayes methods. Biostatistics (Oxford, England). 2007;8(1):118–27. Epub 2006/04/25. doi: 10.1093/biostatistics/kxj037 . 16632515

[105] Hansen KD, Aryee M. IlluminaHumanMethylation450kmanifest: Annotation for Illumina’s 450k methylation arrays. R package version 0.4.0. doi: 10.18129/B9.bioc.IlluminaHumanMethylation450kmanifest 2012.

[106] Tian Y, Morris TJ, Webster AP, Yang Z, Beck S, Feber A, Teschendorff AE. ChAMP: updated methylation analysis pipeline for Illumina BeadChips. Bioinformatics. 2017;33(24):3982–4. doi: 10.1093/bioinformatics/btx513 ; PubMed Central PMCID: PMC5860089. 28961746 PMC5860089

